# Supplementary material for: Three Pairs of Novel Enantiomeric 8-O-4′ Type Neolignans from Saussurea medusa and Their Anti-inflammatory Effects In Vitro
Source: Int J Mol Sci. 2022 Nov 15;23(22):14062. doi: 10.3390/ijms232214062 (PMC9698588; doi:10.3390/ijms232214062)

Supporting Information for

Three Pairs of Novel Enantiomeric 8-*O*-4' Type Neolignans from *Saussurea medusa* and Their Anti-Inflammatory Effect *In Vitro*

Jing-Ya Cao [1,2], Qi Dong [1], Zhi-Yao Wang [3], Li-Juan Mei [1], Yan-duo Tao[1,*] and Rui-Tao Yu[1,*]

Affiliation

[1]Qinghai Provincial Key Laboratory of Tibetan Medicine Research; Key Laboratory of Tibetan Medicine Research, Northwest Institute of Plateau Biology, Chinese Academy of Sciences, Xining 810008, PR China

[2]University of Chinese Academy of Sciences, Beijing 100049, PR China

[3]Henan Academy of Science, Zhengzhou 450002, PR China

Correspondence

Prof. Yan-duo Tao

Northwest Institute of Plateau Biology, Chinese Academy of Sciences, No. 23 Xinning Road, Xining 810008, PR China

E-mail: tyd@nwipb.cas.cn

Prof. Rui-Tao Yu

Northwest Institute of Plateau Biology, Chinese Academy of Sciences, No. 23 Xinning Road, Xining 810008, PR China

Tel: +0971-6143530.

Fax: +0971-614328286.

E-mail: yuruitao@nwipb.cas.cn

Contents

Experimental Section 4

Supplementary References 4

Table S1. Re-optimized conformers, energies and proportions for 8*S*-1 5

Table S2. Re-optimized conformers, energies and proportions for 7*S*,8*S*-2 7

Table S3. Re-optimized conformers, energies and proportions for 7*S*,8*R*-3 9

Figure S1. ^{1}H NMR spectrum of compound 1 (1a/1b) in $CDCl_3$ 13

Figure S2. ^{13}C NMR spectrum of compound 1 (1a/1b) in $CDCl_3$ 14

Figure S3. HSQC spectrum of compound 1 (1a/1b) in $CDCl_3$ 15

Figure S4. HMBC spectrum of compound 1 (1a/1b) in $CDCl_3$ 16

Figure S5. ^{1}H–^{1}H COSY spectrum of compound 1 (1a/1b) in $CDCl_3$ 17

Figure S6. ROESY spectrum of compound 1 (1a/1b) in $CDCl_3$ 18

Figure S7. (+)-ESIMS spectrum of compound 1 (1a/1b) 19

Figure S8. (-)-ESIMS spectrum of compound 1 (1a/1b) 20

Figure S9. (+)-HRESIMS spectrum of compound 1 (1a/1b) 21

Figure S10. IR spectrum of compound 1 (1a/1b) 22

Figure S11. UV spectrum of compound 1 (1a/1b) 23

Figure S12. ^{1}H NMR spectrum of compound 2 (2a/2b) in CD_3OD 24

Figure S13. ^{13}C NMR spectrum of compound 2 (2a/2b) in CD_3OD 25

Figure S14. HSQC spectrum of compound 2 (2a/2b) in CD_3OD 26

Figure S15. HMBC spectrum of compound 2 (2a/2b) in CD_3OD 27

Figure S16. ^{1}H–^{1}H COSY spectrum of compound 2 (2a/2b) in CD_3OD 28

Figure S17. ROESY spectrum of compound 2 (2a/2b) in CD_3OD 29

Figure S18. (+)-ESIMS spectrum of compound 2 (2a/2b) 30

Figure S19. (-)-ESIMS spectrum of compound 2 (2a/2b) 31

Figure S20. (+)-HRESIMS spectrum of compound 2 (2a/2b) 32

Figure S21. IR spectrum of compound 2 (2a/2b) 33

Figure S22. UV spectrum of compound 2 (2a/2b) 34

Figure S23. ^{1}H NMR spectrum of compound 3 (3a/3b) in CD_3OD 35

Figure S24. ^{13}C NMR spectrum of compound 3 (3a/3b) in CD_3OD 36
Figure S25. HSQC spectrum of compound 3 (3a/3b) in CD_3OD 37
Figure S26. HMBC spectrum of compound 3 (3a/3b) in CD_3OD 38
Figure S27. ^{1}H–^{1}H COSY spectrum of compound 3 (3a/3b) in CD_3OD 39
Figure S28. ROESY spectrum of compound 3 (3a/3b) in CD_3OD 40
Figure S29. (+)-ESIMS spectrum of compound 3 (3a/3b) 41
Figure S30. (-)-ESIMS spectrum of compound 3 (3a/3b) 42
Figure S31. (+)-HRESIMS spectrum of compound 3 (3a/3b) 43
Figure S32. IR spectrum of compound 3 (3a/3b) 44
Figure S33. UV spectrum of compound 3 (3a/3b) 45
Figure S34. Chiral HPLC separation profiles of 1a/1b–3a/3b. 46
Figure S35. ^{1}H NMR spectrum of compound 4 in CD_3OD 47
Figure S36. ^{13}C NMR spectrum of compound 4 in CD_3OD 48
Figure S37. ^{1}H NMR spectrum of compound 5 in CD_3OD 49
Figure S38. ^{13}C NMR spectrum of compound 5 in CD_3OD 50
Figure S39. ^{1}H NMR spectrum of compound 6 in CD_3OD 51
Figure S40. ^{13}C NMR spectrum of compound 6 in CD_3OD 52
Figure S41. ^{1}H NMR spectrum of compound 7 in $CDCl_3$ 53
Figure S42. ^{13}C NMR spectrum of compound 7 in $CDCl_3$ 54
Figure S43. ^{1}H NMR spectrum of compound 8 in $CDCl_3$ 55
Figure S44. ^{13}C NMR spectrum of compound 8 in $CDCl_3$ 56
Figure S45. ^{1}H NMR spectrum of compound 9 in $CDCl_3$ 57
Figure S46. ^{13}C NMR spectrum of compound 9 in $CDCl_3$ 58
Figure S47. ^{1}H NMR spectrum of compound 10 in $CDCl_3$ 59
Figure S48. ^{13}C NMR spectrum of compound 10 in $CDCl_3$ 60

Experimental Section

*ECD calculations for **1a/1b−3a/3b***

The absolute configurations of **1a/1b−3a/3b** were determined by quantum chemical TDDFT calculations of their theoretical ECD spectra. Using the MM2 force field in the Chem3D pro 14.0 software, the initial conformers of each compound were established. Conformational searches were conducted with the torsional sampling method (Monte Carlo Multiple Minimum, MCMM) under OPLS3 [1] force field by Maestro 11.5 software (Maestro Technologies, Inc., Trenton, NJ, USA) in an energy window of 12.6 kJ/mol. The conformational optimization and the following TDDFT calculations for the conformers that satisfied the experiment coupling constants and NOE signals were all carried out with the Gaussian 16 program package [2] at the B3LYP/6-31G(d) level in methanol. All TDDFT calculations were computed at the PCM/*ω*B97XD/6-311G** level of theory in methanol. Finally, the Boltzmann-averaged ECD spectra were simulated with SpecDis 1.71 [3,4].

Supplementary References

1. Harder, E.; Damm, W.; Maple, J.; Wu, C.J.; Reboul, M.; Xiang, J.Y.; Wang, L.L.; Lupyan, D.; Dahlgren, M.K.; Knight, J.L.; Kaus, J.W.; Cerutti, D.; Krilov, G.; Jorgensen, W.L.; Abel, R.; Friesner, R.A. OPLS3: A force field providing broad coverage of drug-like small molecules and proteins. *J. Chem. Theory Comput.* **2015**, *12*, 281–296.
2. Frisch, M.J.; Trucks, G.W.; Schlegel, H.B.; Scuseria, G.E.; Robb, M.A.; Cheeseman, J.R.; Scalmani, G.; Barone, B.V.; Petersson, G.A.; Nakatsuji, H.; Li, X.; Caricato, M.; Marenich, A.V.; Bloino, J.; Janesko, B.G.; Gomperts, R.; Mennucci, B.; Hratchian, H.P.; Ortiz, J.V.; Izmaylov, A.F.; Sonnenberg, J.L.;

Williams, D.; Ding, F.; Lipparini, F.; Egidi, F.; Goings, J.; Peng, B.; Petrone, A.; Henderson, T.; Ranasinghe, D.; Zakrzewski, V.G.; Gao, J.; Rega, N.; Zheng, G.; Liang, W.; Hada, M.; Ehara, M.; Toyota, K.; Fukuda, R.; Hasegawa, J.; Ishida, M.; Nakajima, T.; Honda, Y.; Kitao, O.; Nakai, H.; Vreven, T.; Throssell, K.; Peralta, J.E.; Ogliaro, F.; Bearpark, M.J.; Heyd, J.J.; Brothers, E.N.; Kudin, K.N.; Staroverov, V.N.; Keith, T.A.; Kobayashi, R.; Normand, J.; Raghavachari, K.; Rendell, A.P.; Burant, J.C.; Iyengar, S.S.; Tomasi, J.; Cossi, M.; Millam, J.M.; Klene, M.; Adamo, C.; Cammi, R.; Ochterski, J.W.; Martin, R.L.; Morokuma, K.; Farkas, O.; Foresman, J.B.; Fox, D.J. 2016. Gaussian 16 Rev. A.03, Wallingford, CT.

3. Bruhn, T.; Schaumlöffel, A.; Hemberger, Y.; Bringmann, G. SpecDis: Quantifying the comparison of calculated and experimental electronic circular dichroism spectra. *Chirality* **2013**, *25*, 243–249.
4. Pescitelli, G.; Bruhn, T. Good computational practice in the assignment of absolute configurations by TDDFT calculations of ECD spectra. *Chirality* **2016**, *28*, 466–474.

Table S1. Re-optimized conformers, energies and proportions for 8*S*-1

Number	Conformer	Energy (hartree)	Energy (Kcal/mol)	Proportion (%)
1	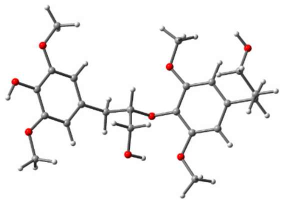	-1457.813011	-914791.3547	18.87

2	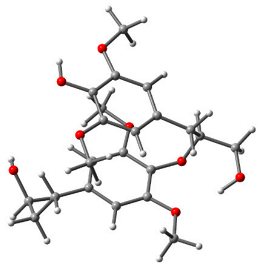	-1457.812988	-914791.3403	18.41
3	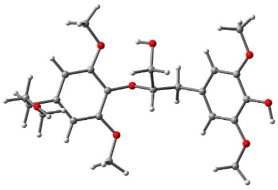	-1457.812494	-914791.0303	10.90
4	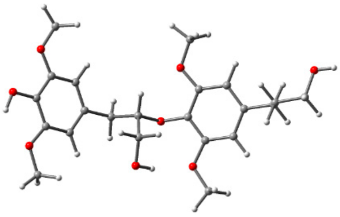	-1457.812199	-914790.8452	7.98
5	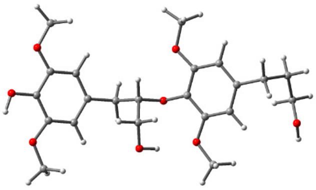	-1457.812192	-914790.8408	7.92
6	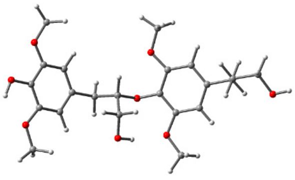	-1457.811999	-914790.7197	6.45
7	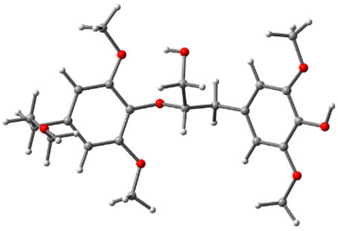	-1457.811909	-914790.6632	5.86
8	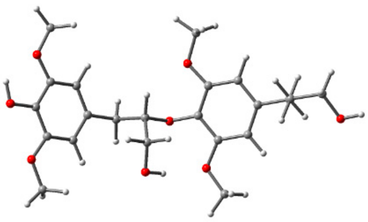	-1457.81177	-914790.576	5.06

9	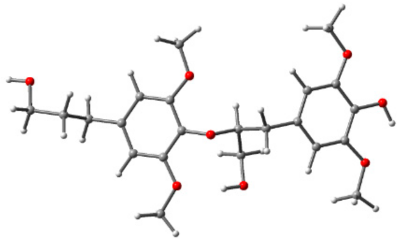	-1457.811683	-914790.5214	4.61
10	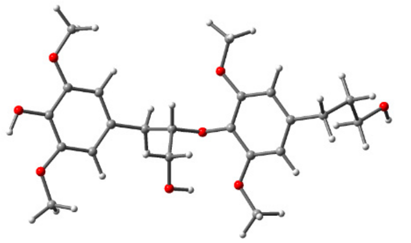	-1457.811632	-914790.4894	4.37
11	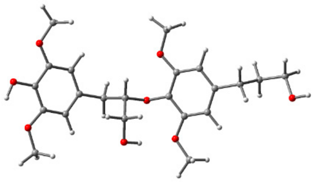	-1457.811613	-914790.4775	4.28
12	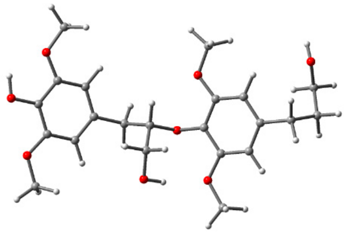	-1457.811479	-914790.3934	3.72
13	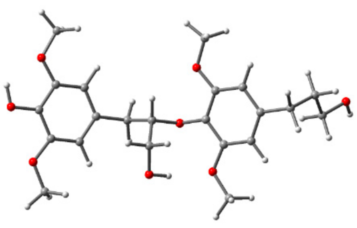	-1457.810661	-914789.8801	1.56

Table S2. Re-optimized conformers, energies and proportions for 7*S*,8*S*-2

Number	Conformer	Energy (hartree)	Energy (Kcal/mol)	Proportion (%)

1	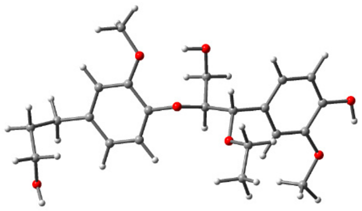	-1382.623515	-867609.2399	23.79
2	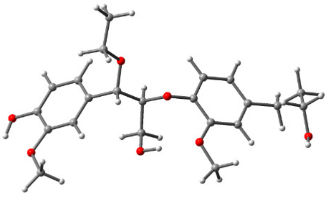	-1382.623205	-867609.0454	17.12
3	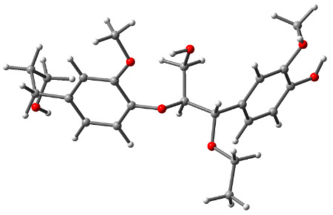	-1382.622998	-867608.9155	13.75
4	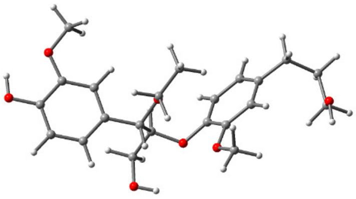	-1382.622808	-867608.7962	11.24
5	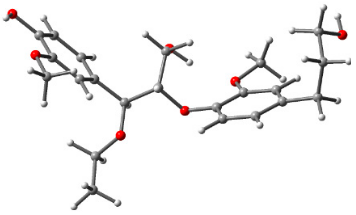	-1382.62275	-867608.7598	10.57
6	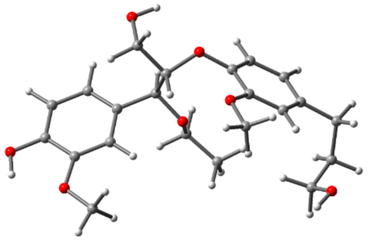	-1382.622612	-867608.6732	9.13
7	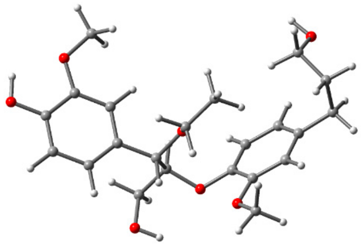	-1382.622256	-867608.4498	6.26

8	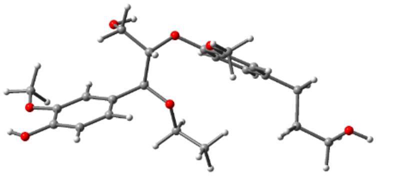	-1382.621529	-867607.9936	2.90
9	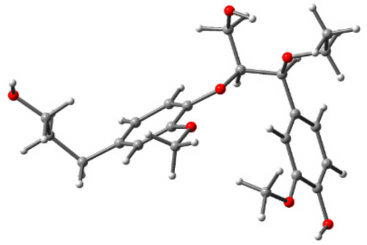	-1382.621204	-867607.7897	2.05
10	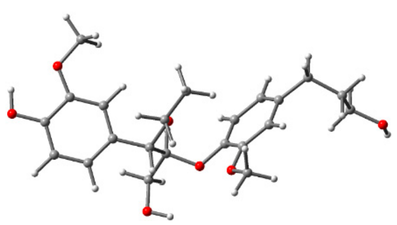	-1382.621056	-867607.6968	1.75
11	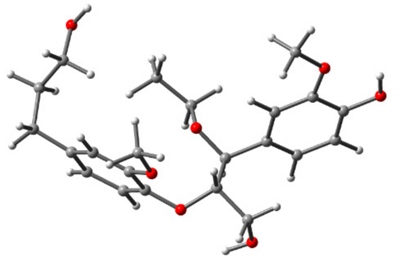	-1382.620513	-867607.3561	0.99
12	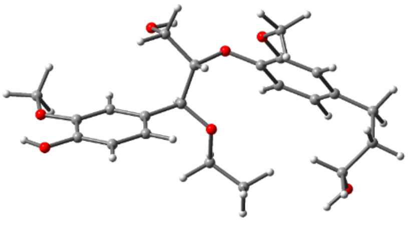	-1382.619755	-867606.8804	0.44

Table S3. Re-optimized conformers, energies and proportions for 7*S*,8*R*-3

Number	Conformer	Energy (hartree)	Energy (Kcal/mol)	Proportion (%)

1	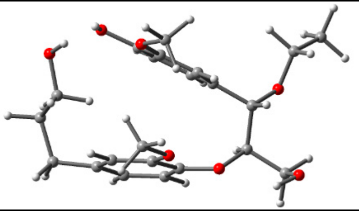	-1382.627683	-867611.8553	60.13
2	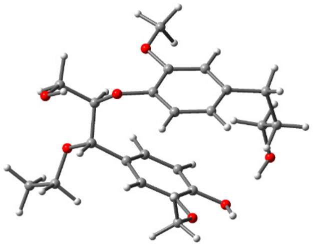	-1382.626968	-867611.4067	28.17
3	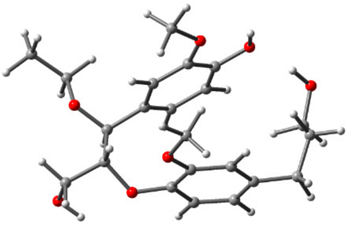	-1382.62594	-867610.7616	9.47
4	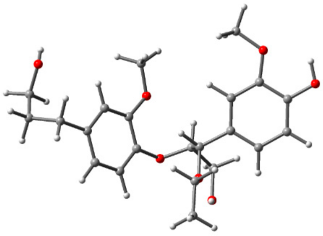	-1382.623581	-867609.2813	0.78
5	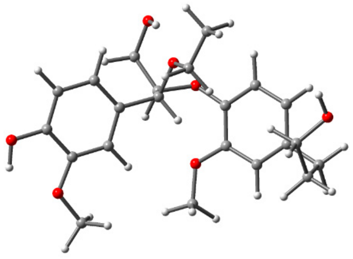	-1382.623403	-867609.1696	0.64
6	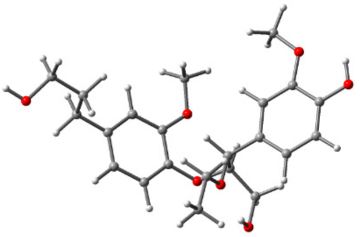	-1382.622399	-867608.5396	0.22
7	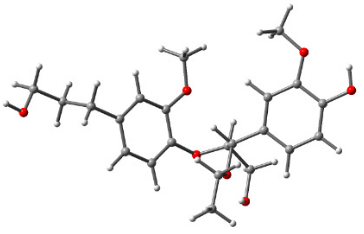	-1382.622272	-867608.4599	0.19

8	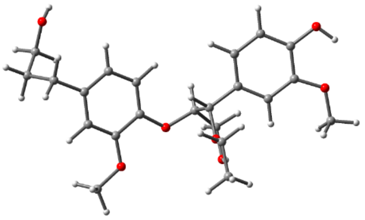	-1382.621888	-867608.2189	0.13
9	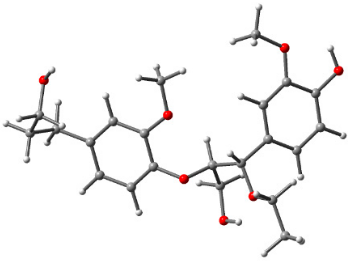	-1382.62164	-867608.0633	0.10
10	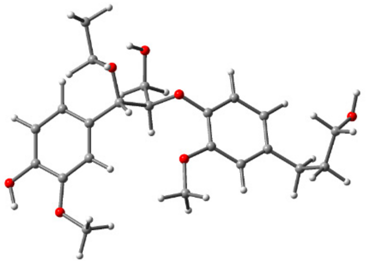	-1382.621119	-867607.7364	0.06
11	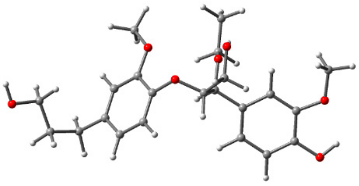	-1382.620822	-867607.55	0.04
12	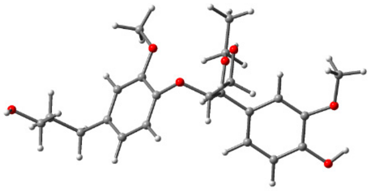	-1382.620534	-867607.3693	0.03
13	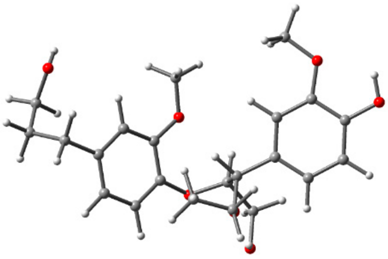	-1382.619476	-867606.7054	0.01
14	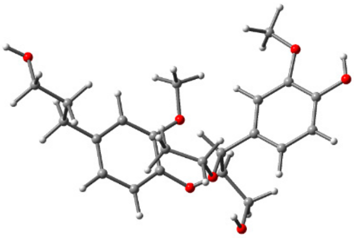	-1382.619318	-867606.6062	0.01

15	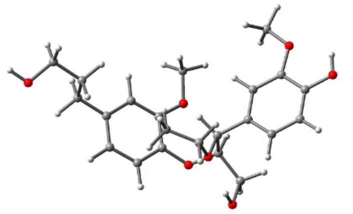	-1382.619302	-867606.5962	0.01
16	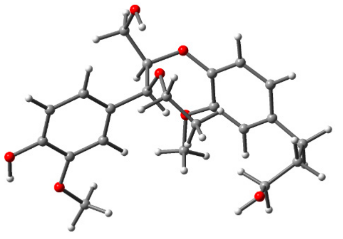	-1382.618206	-867605.9084	0.01

Figure S1. ^{1}H NMR spectrum of compound 1 (1a/1b) in $CDCl_3$

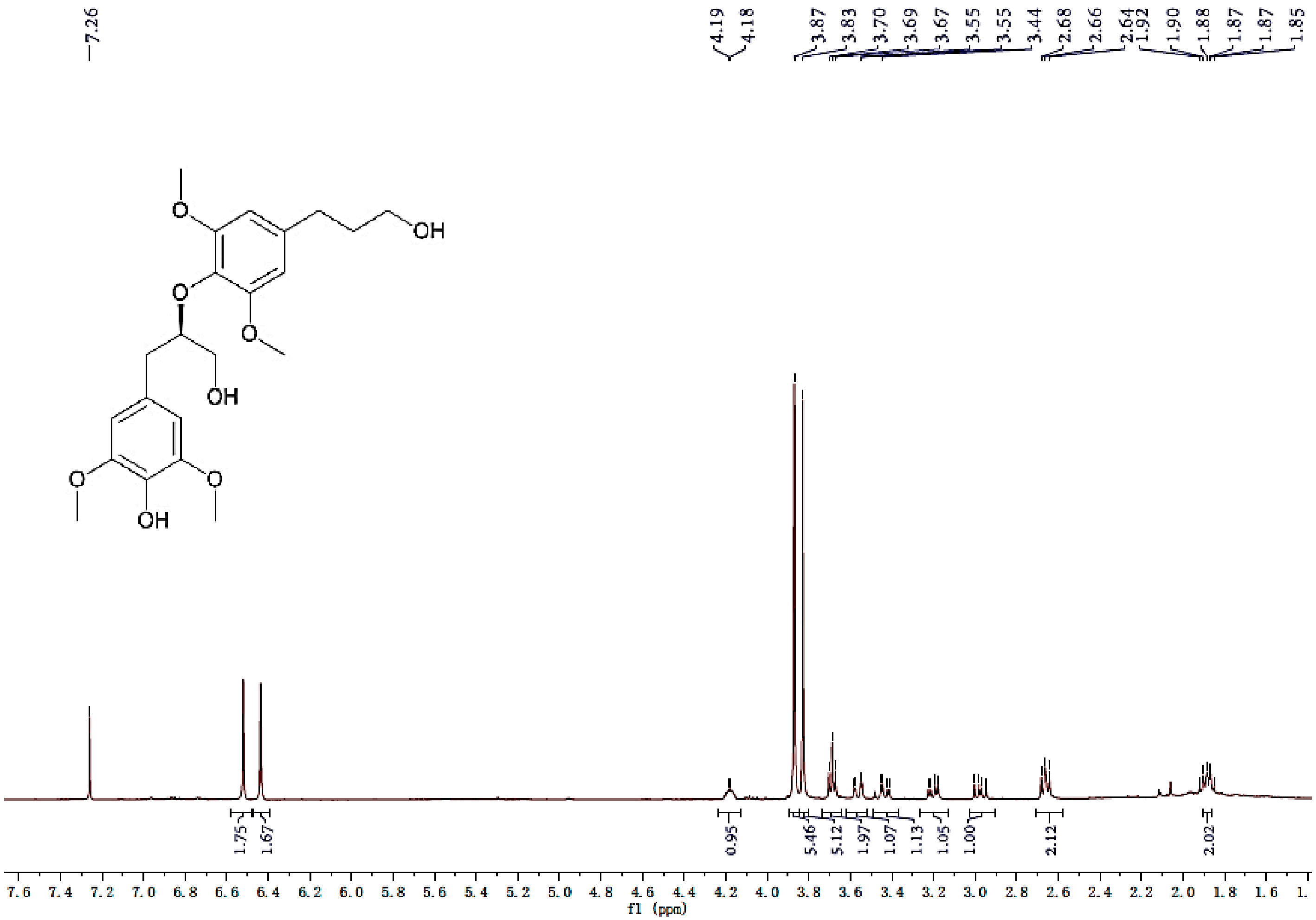

Figure S2. ^{13}C NMR spectrum of compound 1 (1a/1b) in $CDCl_3$

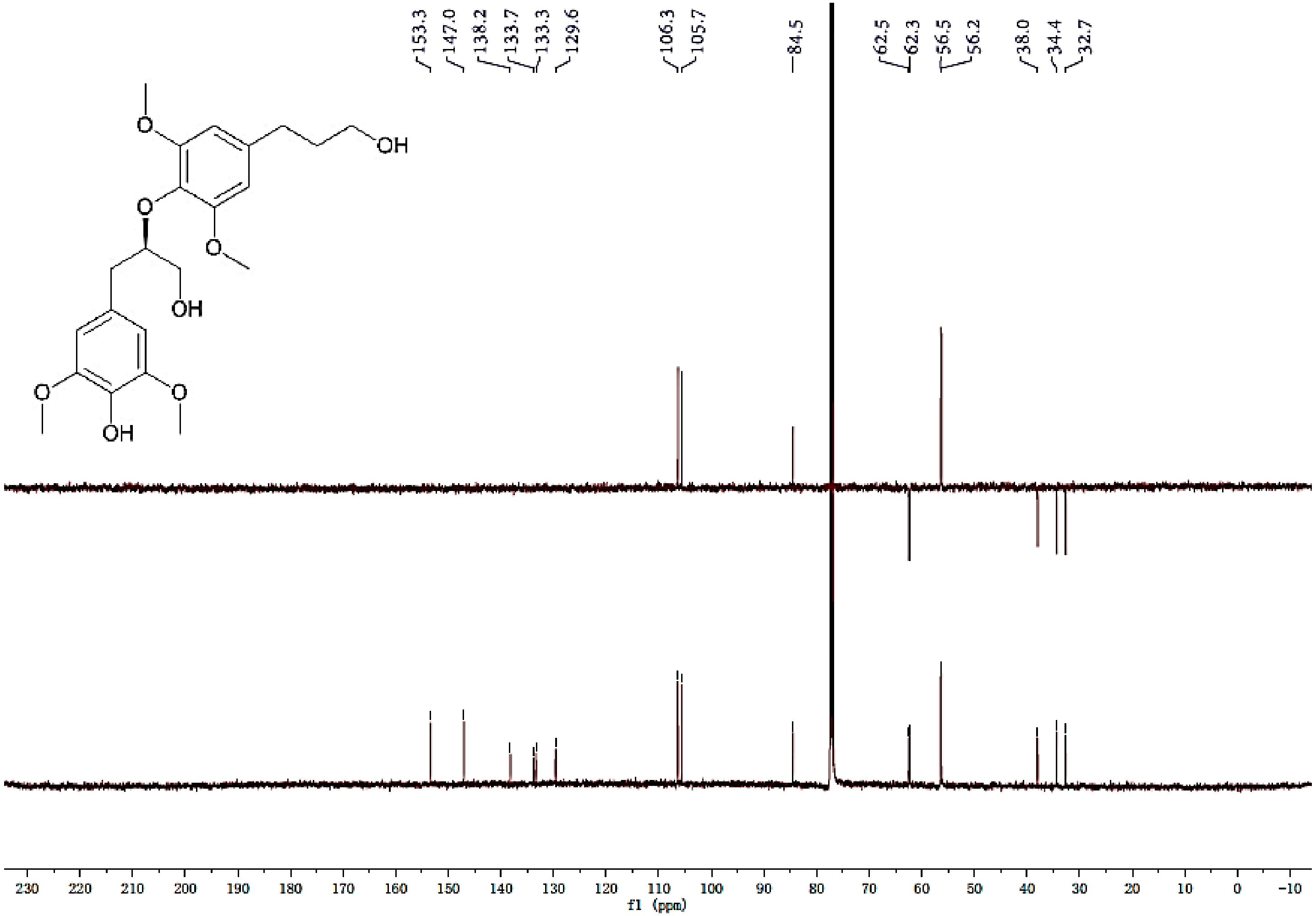

Figure S3. HSQC spectrum of compound 1 (1a/1b) in $CDCl_3$

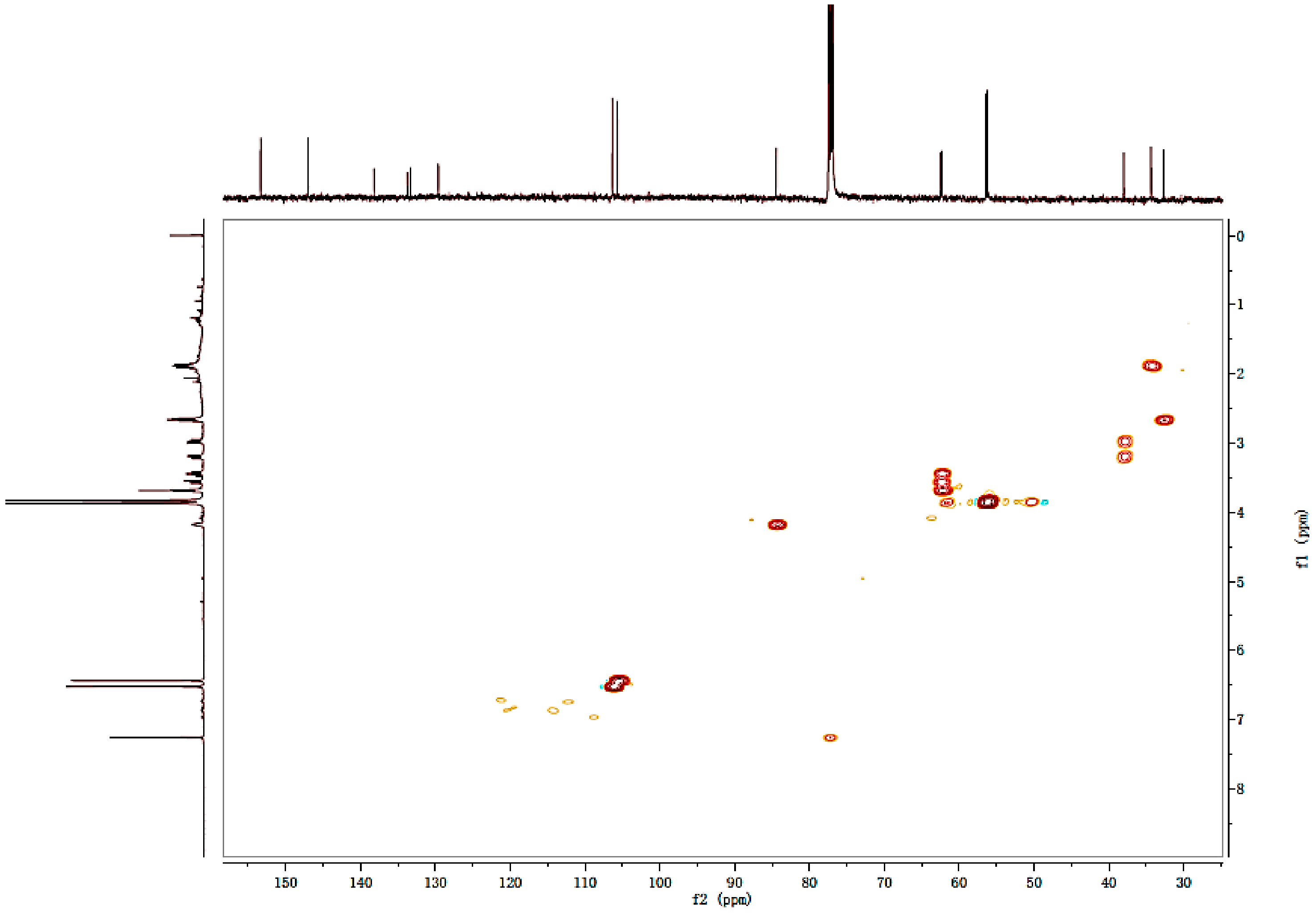

Figure S4. HMBC spectrum of compound 1 (1a/1b) in $CDCl_3$

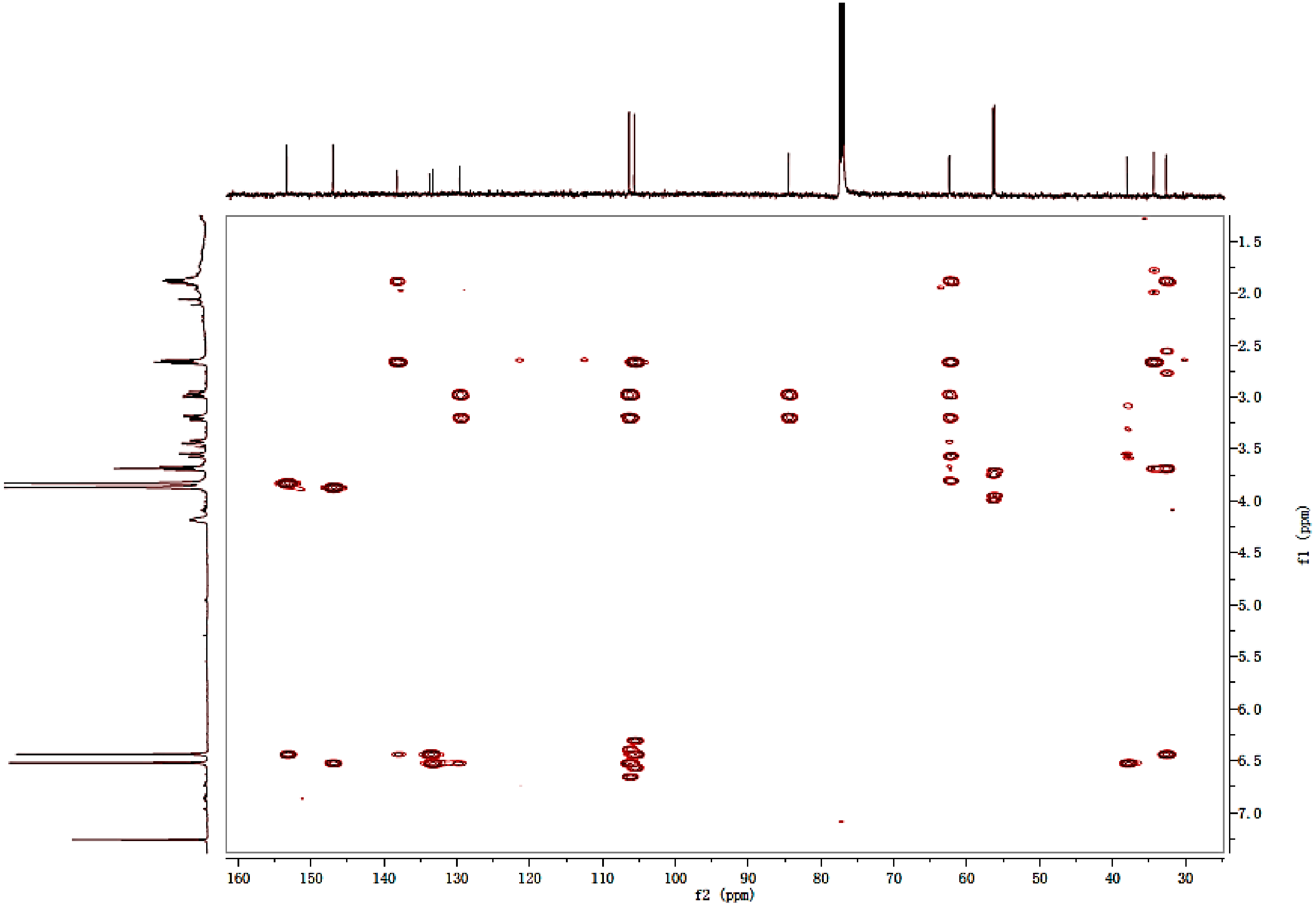

Figure S5. ^{1}H–^{1}H COSY spectrum of compound 1 (1a/1b) in $CDCl_3$

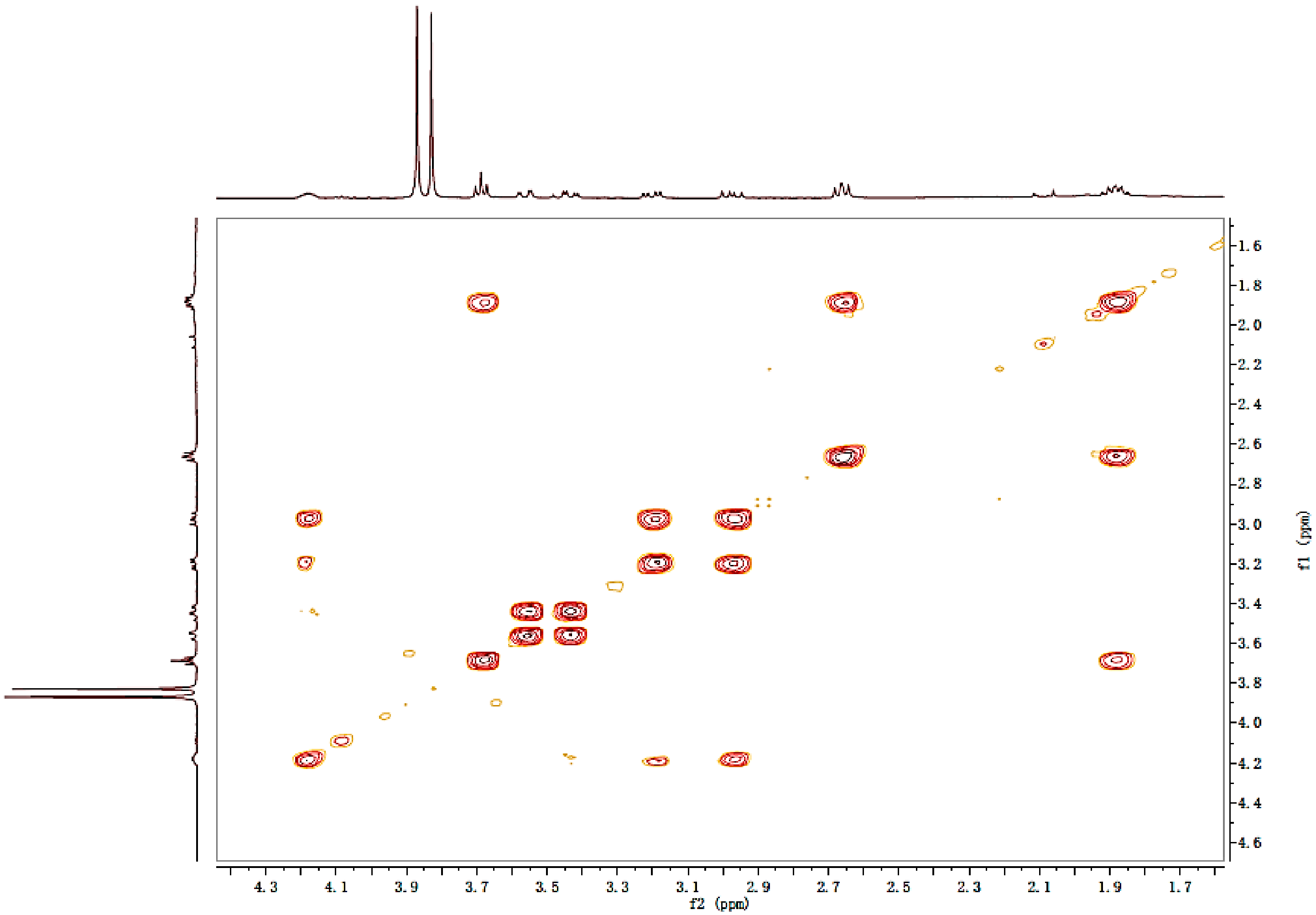

Figure S6. ROESY spectrum of compound 1 (1a/1b) in $CDCl_3$

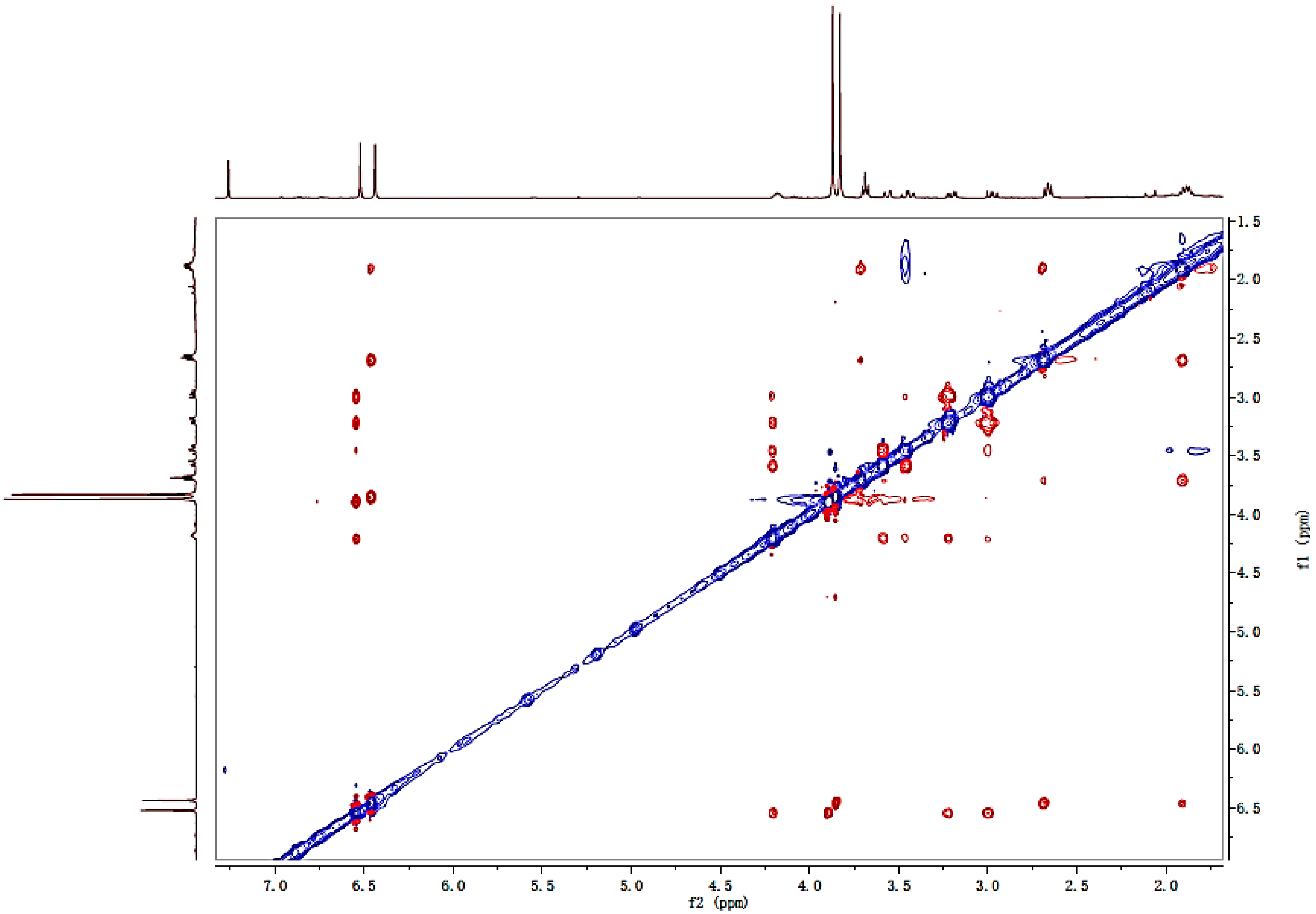

Figure S7. (+)-ESIMS spectrum of compound 1 (1a/1b)

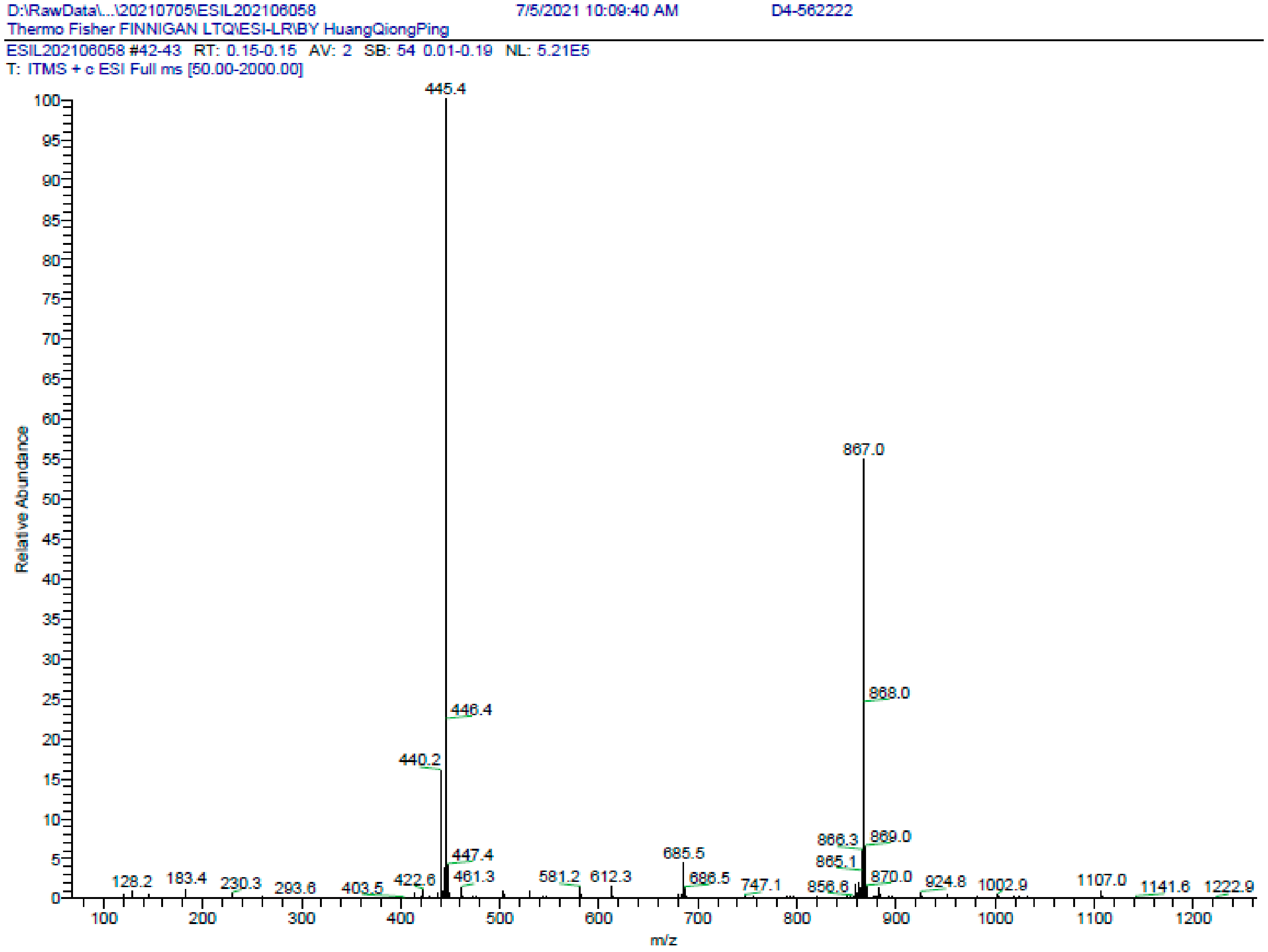

Figure S8. (-)-ESIMS spectrum of compound 1 (1a/1b)

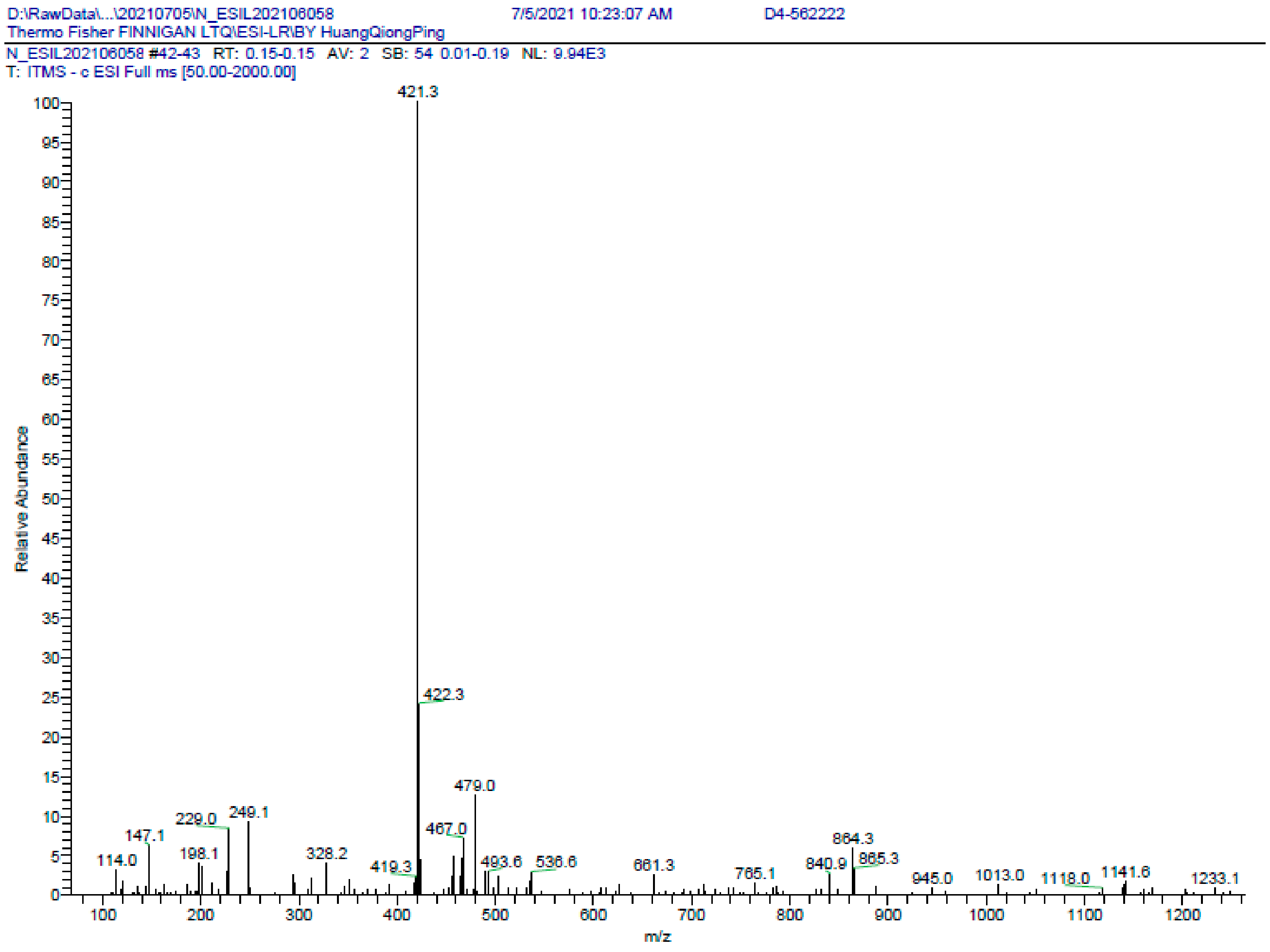

Figure S9. (+)-HRESIMS spectrum of compound 1 (1a/1b)

Qualitative Analysis Report

**Data Filename**	ESIH202103316.d	**Sample Name**	D4-562222
**Sample ID**		**Position**	P1-C5
**Instrument Name**	Agilent G6520 Q-TOF	**Acq Method**	20160322_MS_ESIH_POS_1min.m
**Acquired Time**	7/5/2021 20:49:41	**IRM Calibration Status**	Success
**DA Method**	small molecular data analysis method.m	**Comment**	ESIH by zhuzhenyun

User Spectra

Fragmentor Voltage	Collision Energy	Ionization Mode
135	0	ESI

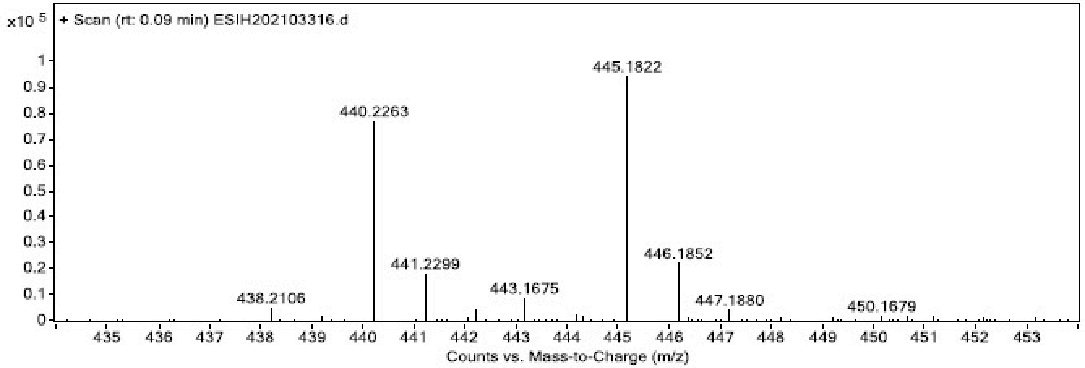

Formula Calculator Results

m/z	Calc m/z	Diff (mDa)	Diff (ppm)	Ion Formula	Ion
443.1675	443.1676	0.13	0.29	C22 H28 Na O8	(M+Na)+
445.1822	445.1833	1.05	2.35	C22 H30 Na O8	(M+Na)+

--- End Of Report ---

Figure S10. IR spectrum of compound 1 (1a/1b)

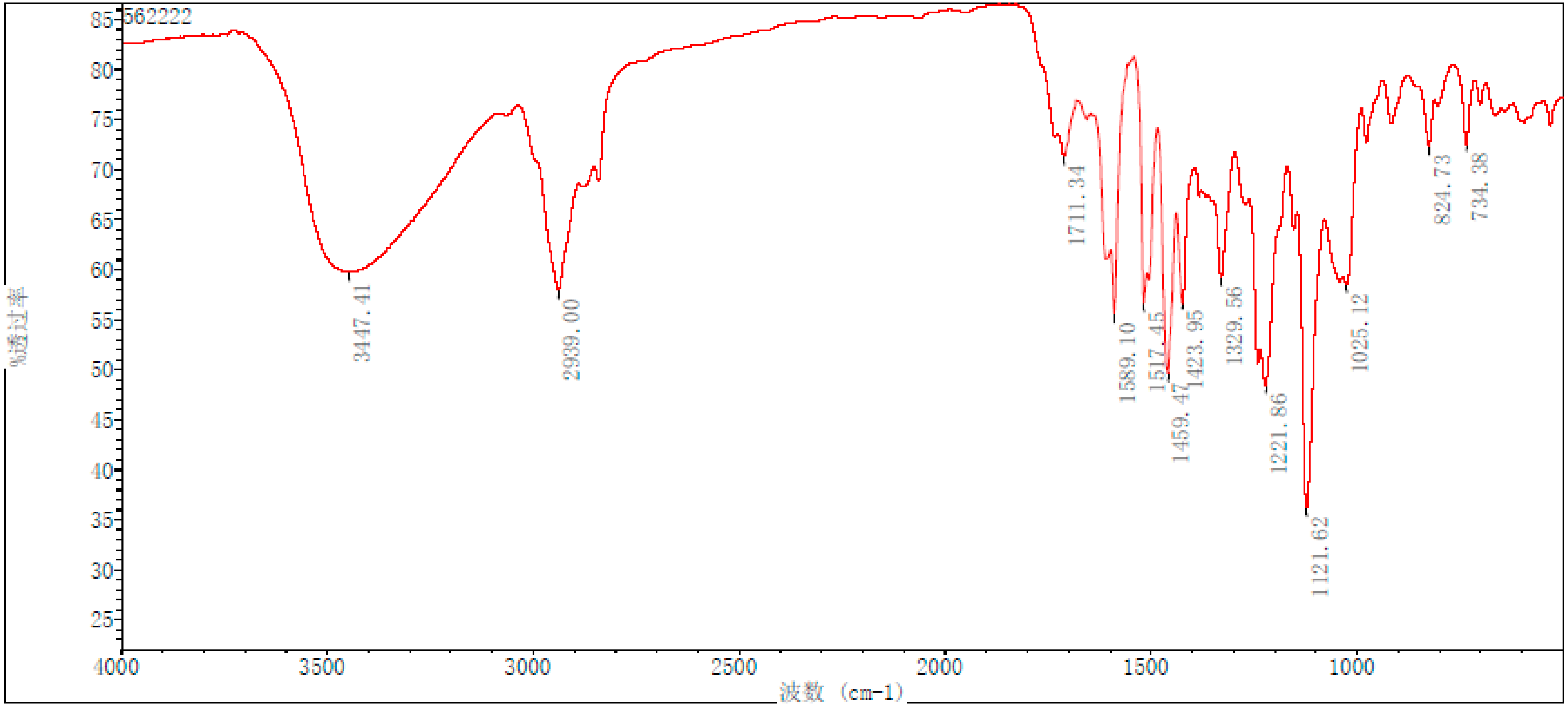

Figure S11. UV spectrum of compound 1 (1a/1b)

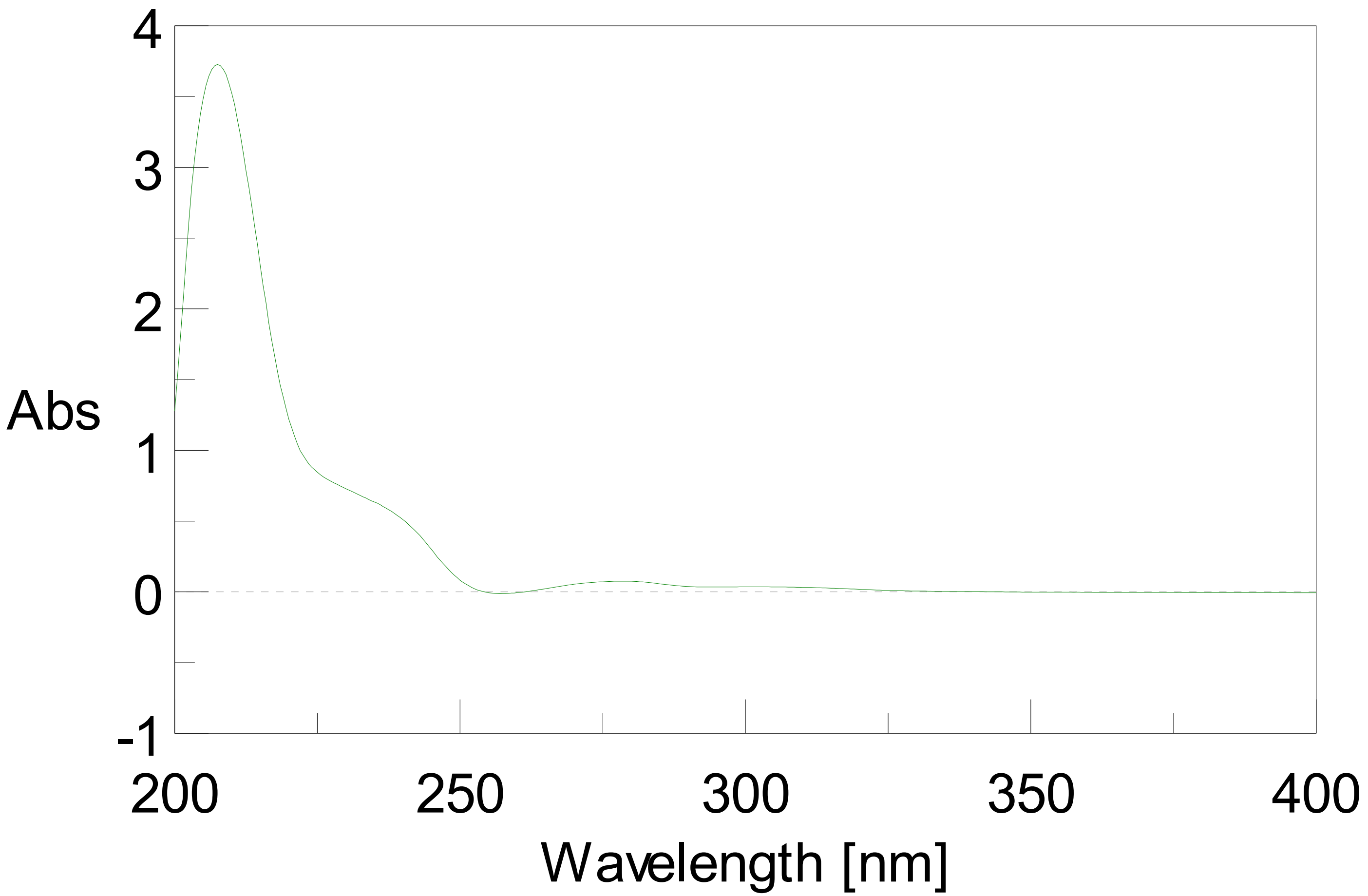

Figure S12. ^{1}**H NMR spectrum of compound 2 (2a/2b) in CD$_3$OD**

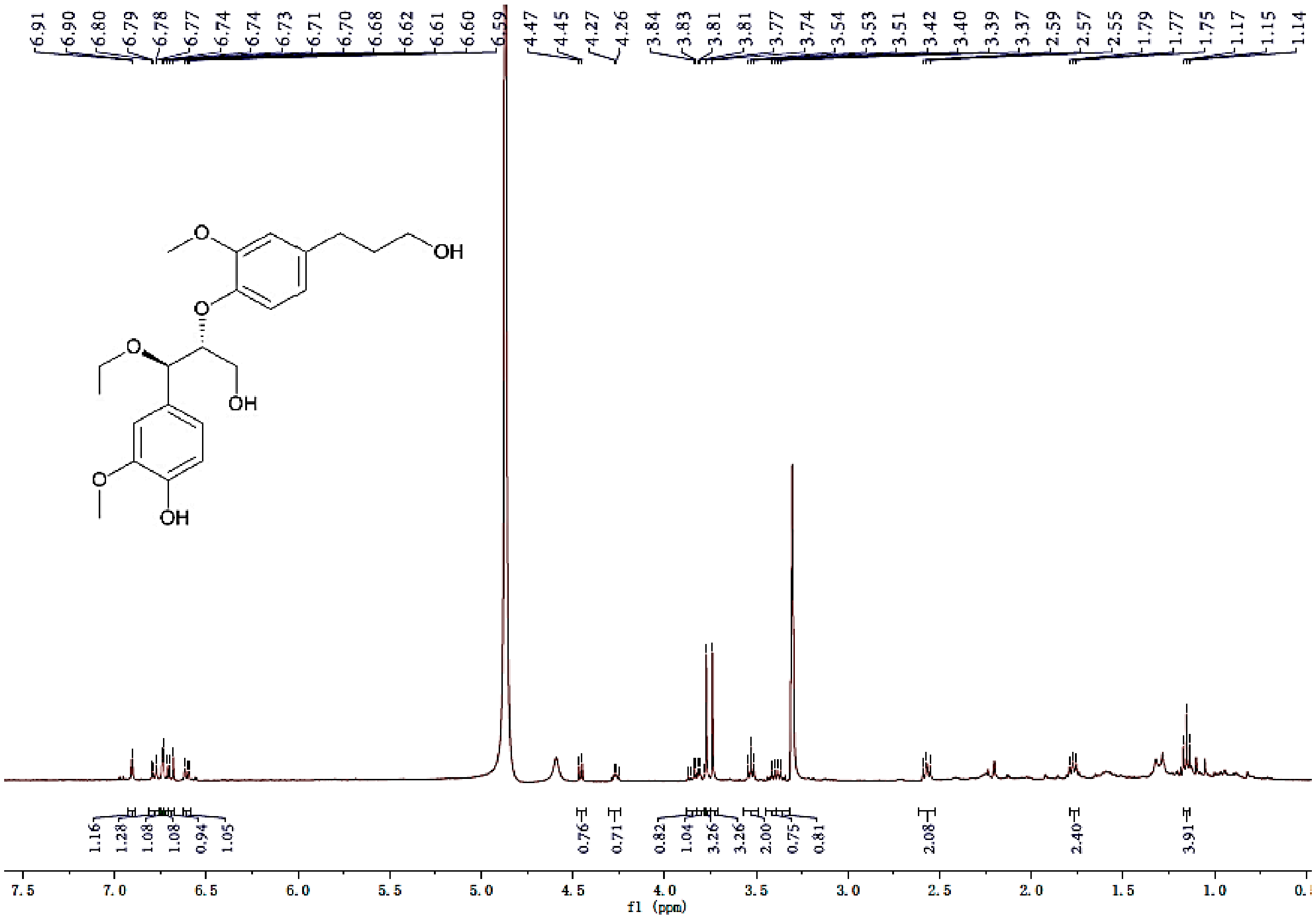

Figure S13. ^{13}C NMR spectrum of compound 2 (2a/2b) in CD_3OD

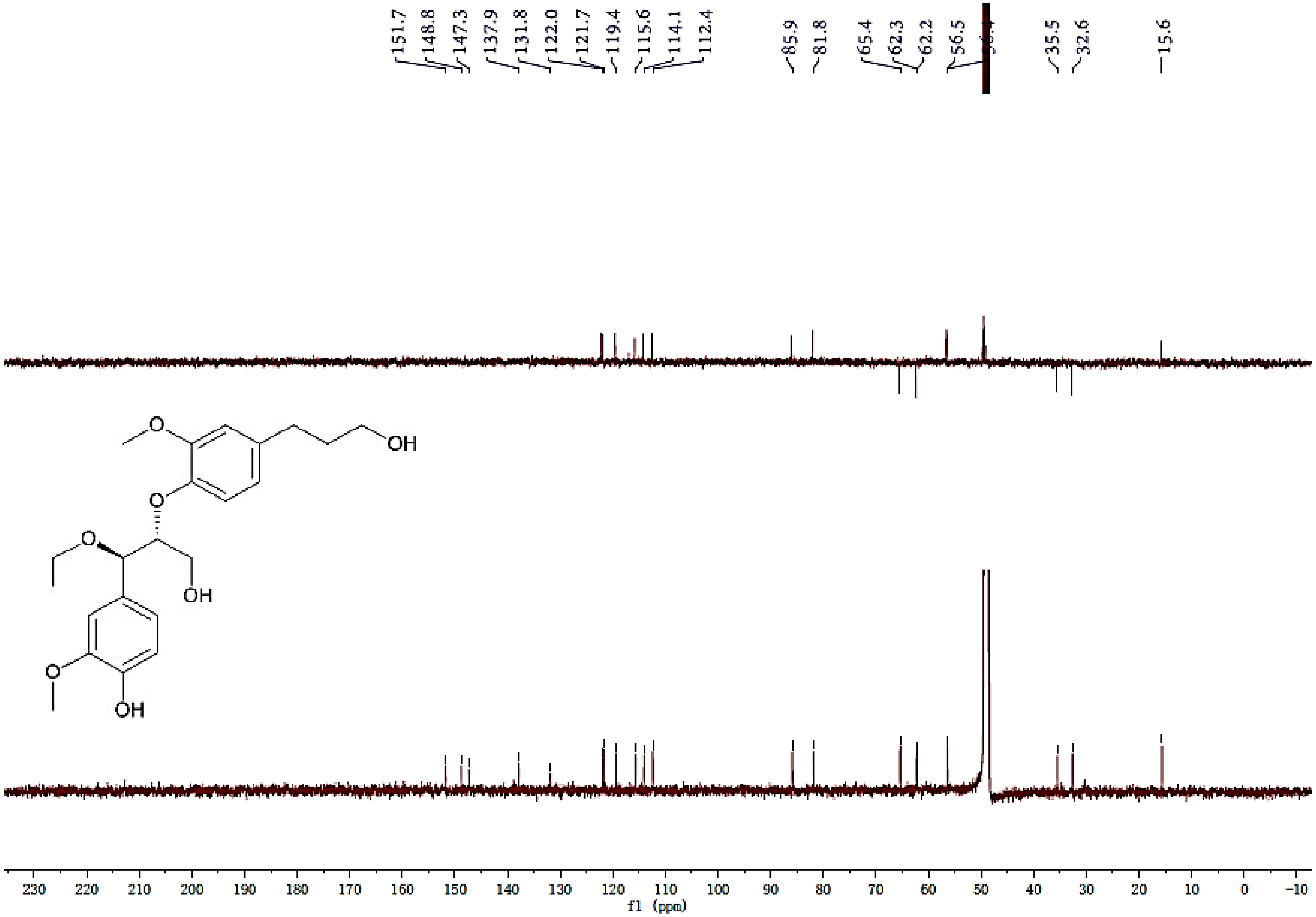

Figure S14. HSQC spectrum of compound 2 (2a/2b) in CD_3OD

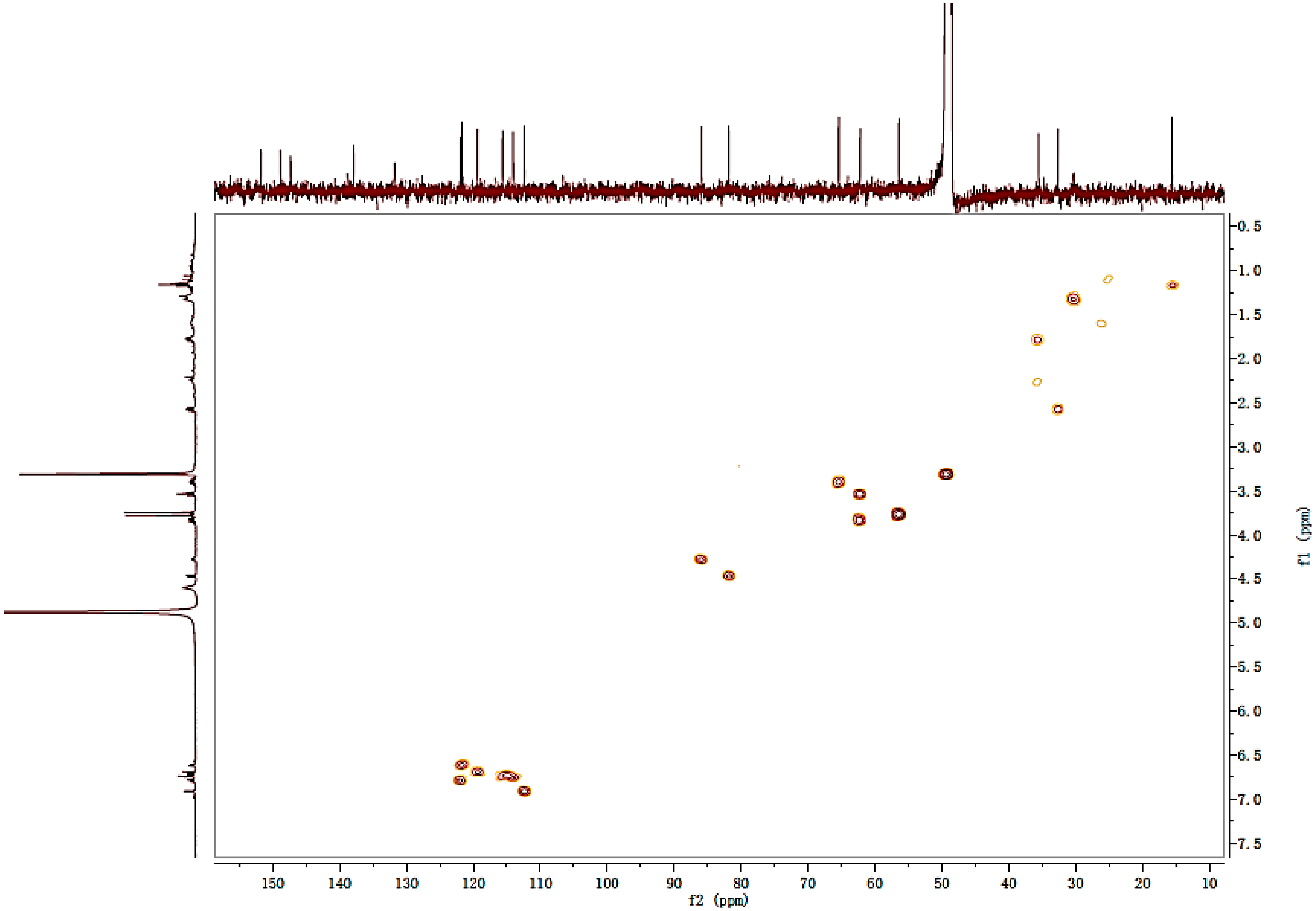

Figure S15. HMBC spectrum of compound 2 (2a/2b) in CD_3OD

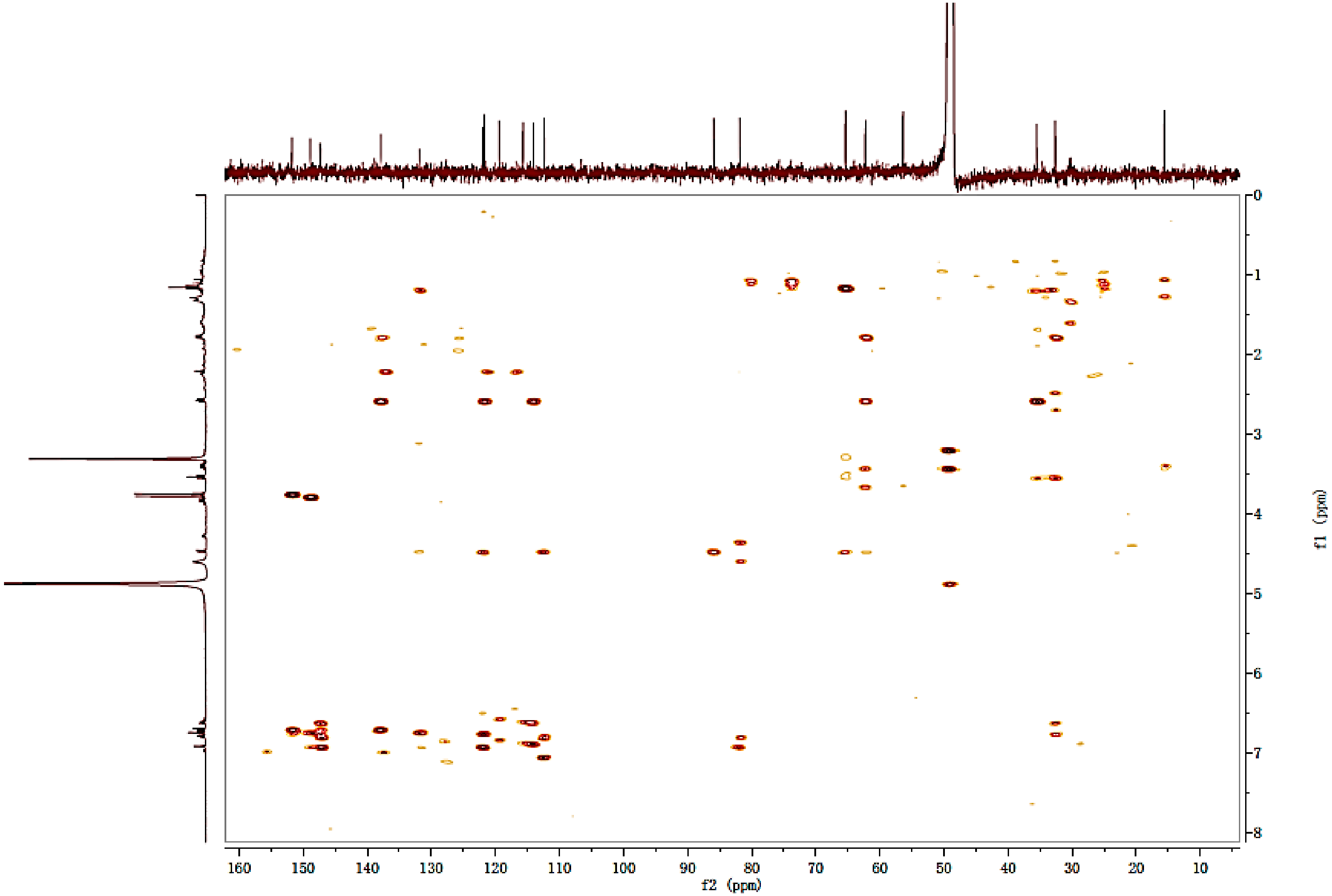

Figure S16. ^{1}H–^{1}H COSY spectrum of compound 2 (2a/2b) in CD_3OD

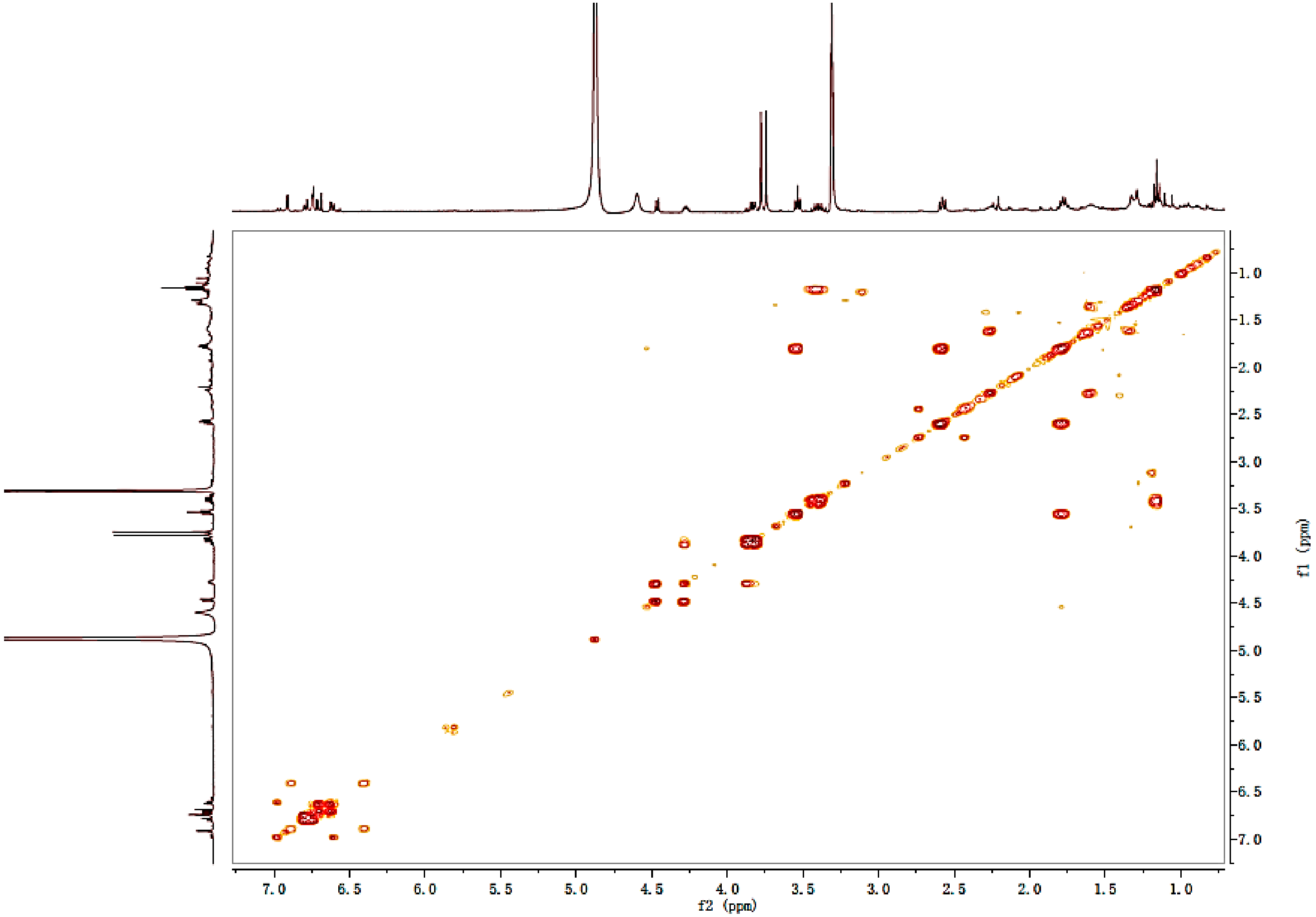

Figure S17. ROESY spectrum of compound 2 (2a/2b) in CD_3OD

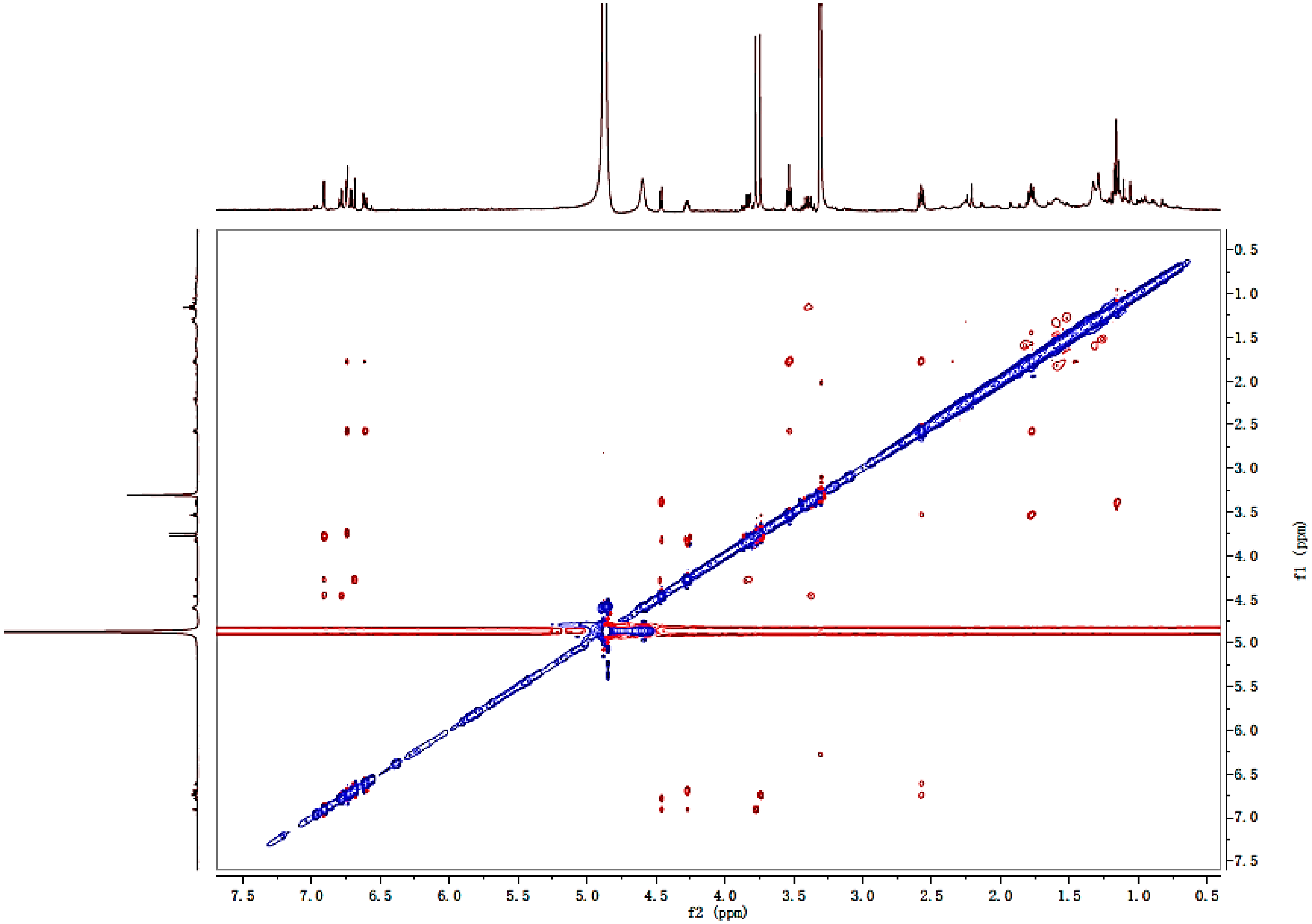

Figure S18. (+)-ESIMS spectrum of compound 2 (2a/2b)

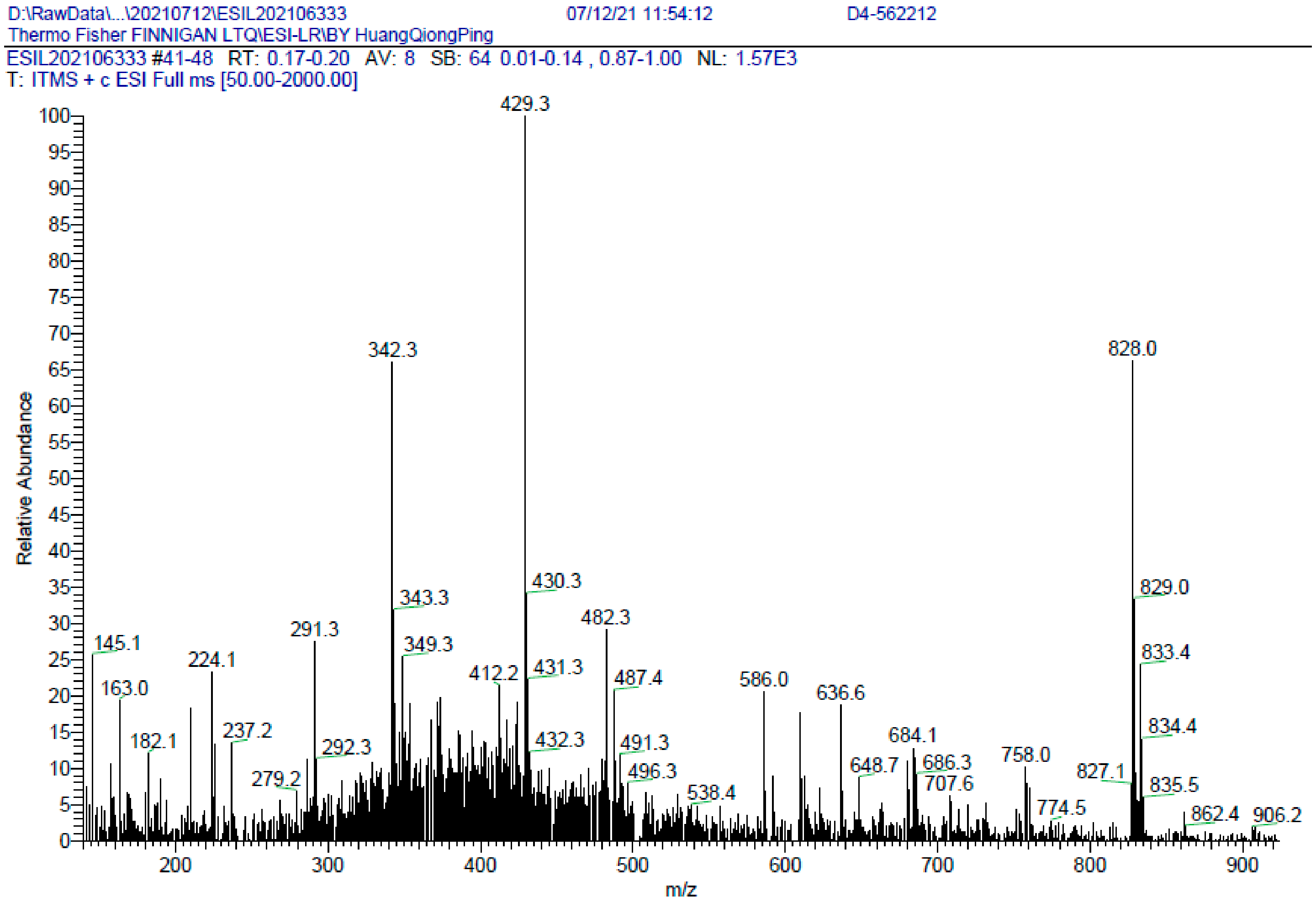

Figure S19. (-)-ESIMS spectrum of compound 2 (2a/2b)

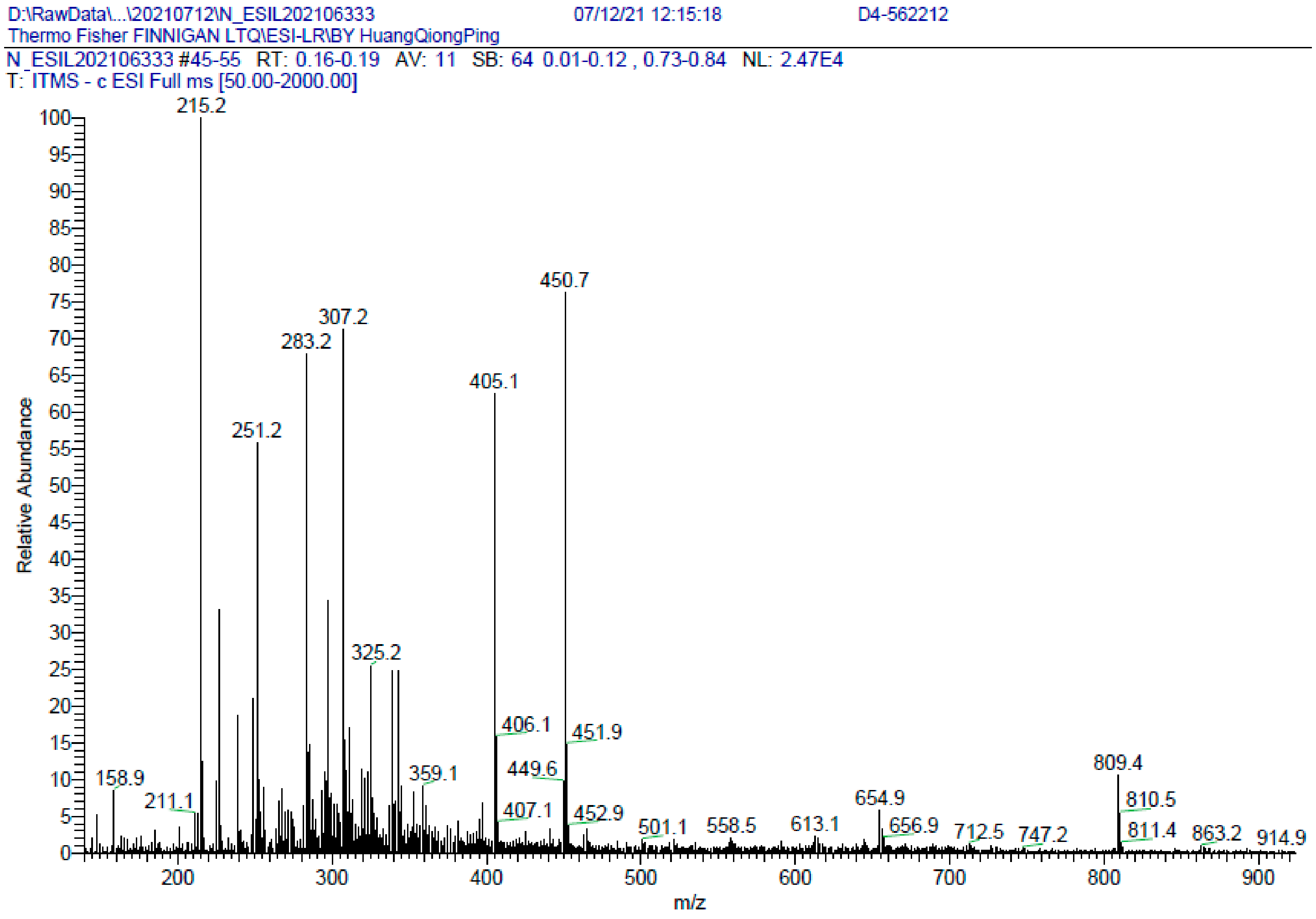

Figure S20. (+)-HRESIMS spectrum of compound 2 (2a/2b)

Qualitative Analysis Report

**Data Filename**	ESIH202103425.d	**Sample Name**	D4-562212
**Sample ID**		**Position**	P1-A6
**Instrument Name**	Agilent G6520 Q-TOF	**Acq Method**	20160322_MS_ESIH_POS_1min.m
**Acquired Time**	7/12/2021 18:14:40	**IRM Calibration Status**	Success
**DA Method**	small molecular data analysis method.m	**Comment**	ESIH by zhuzhenyun

User Spectra

Fragmentor Voltage	Collision Energy	Ionization Mode
135	0	ESI

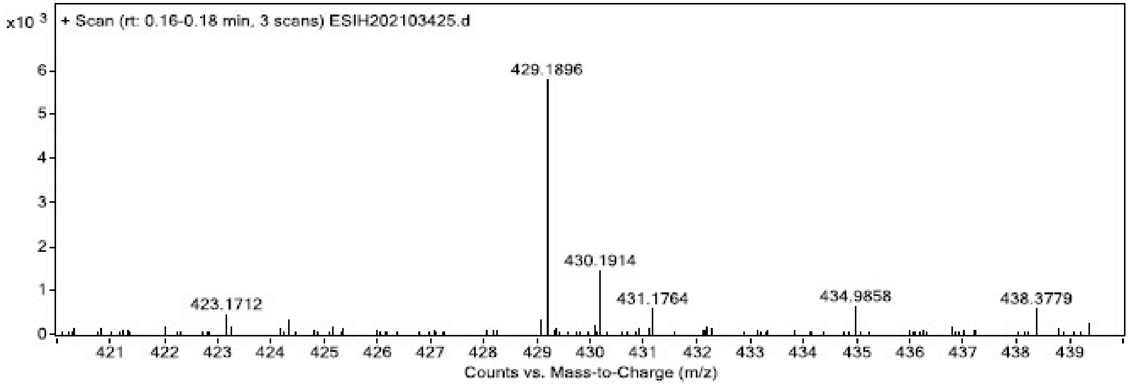

Formula Calculator Results

m/z	Calc m/z	Diff (mDa)	Diff (ppm)	Ion Formula	Ion
429.1896	429.1884	-1.18	-2.76	C22 H30 Na O7	(M+Na)+

--- End Of Report ---

Figure S21. IR spectrum of compound 2 (2a/2b)

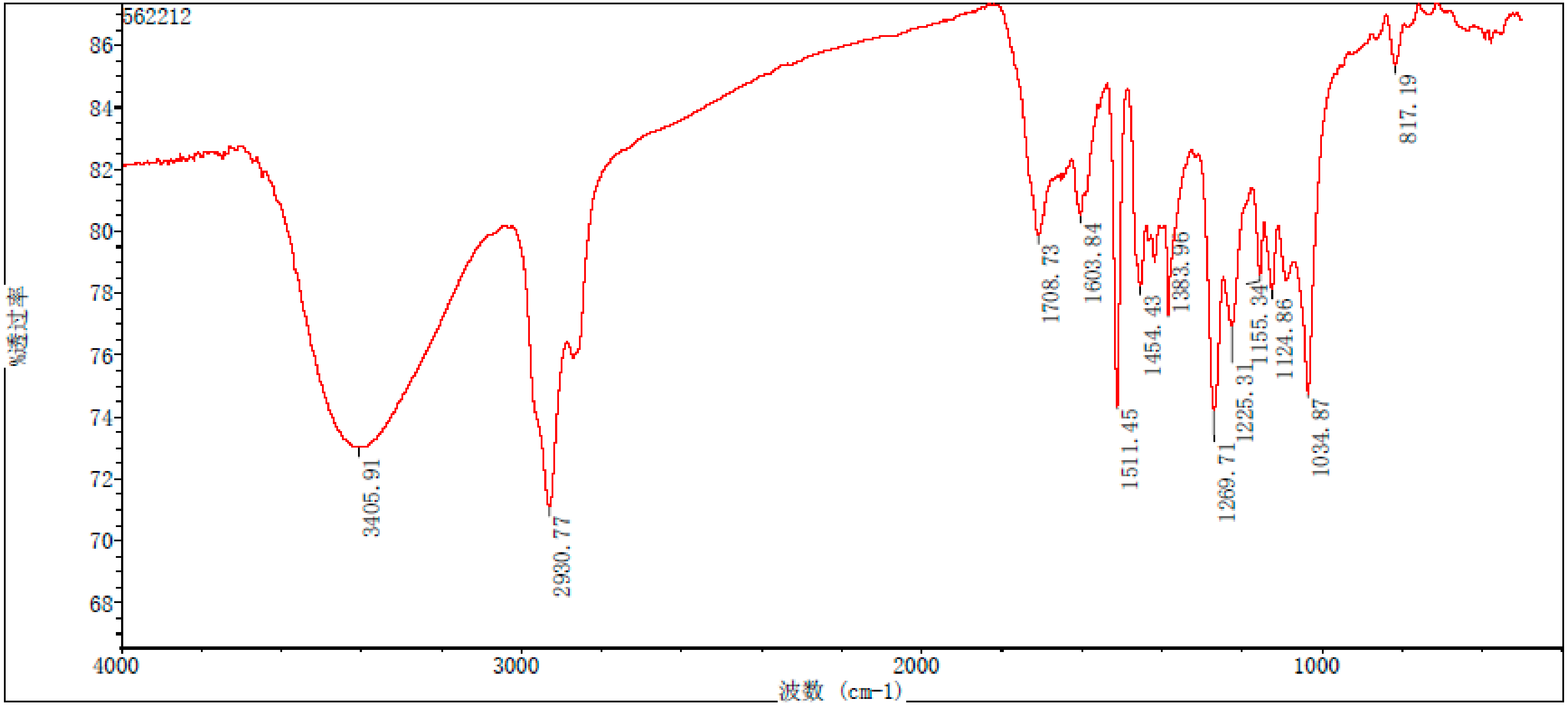

Figure S22. UV spectrum of compound 2 (2a/2b)

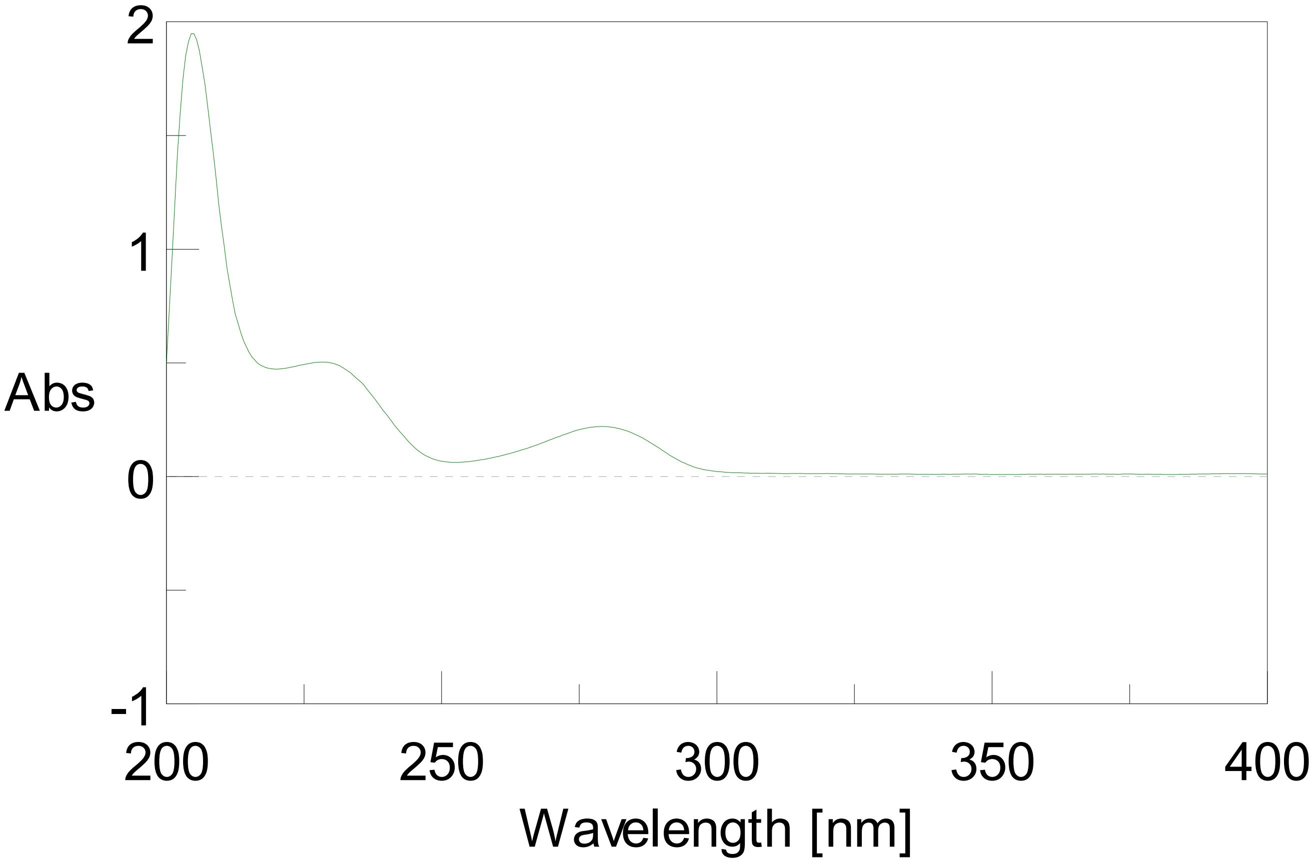

Figure S23. ^{1}H NMR spectrum of compound 3 (3a/3b) in CD_3OD

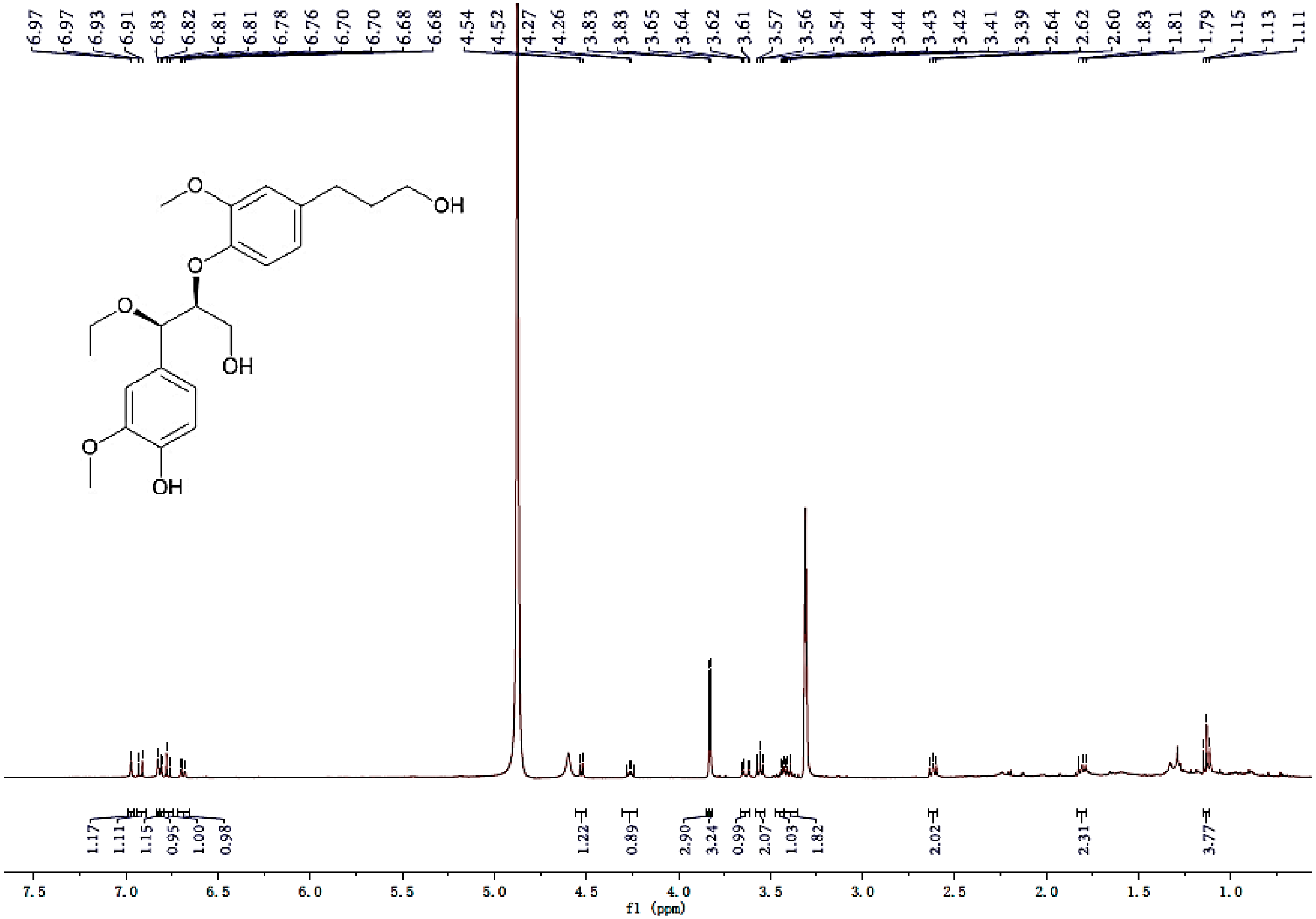

Figure S24. ^{13}**C NMR spectrum of compound 3 (3a/3b) in CD$_3$OD**

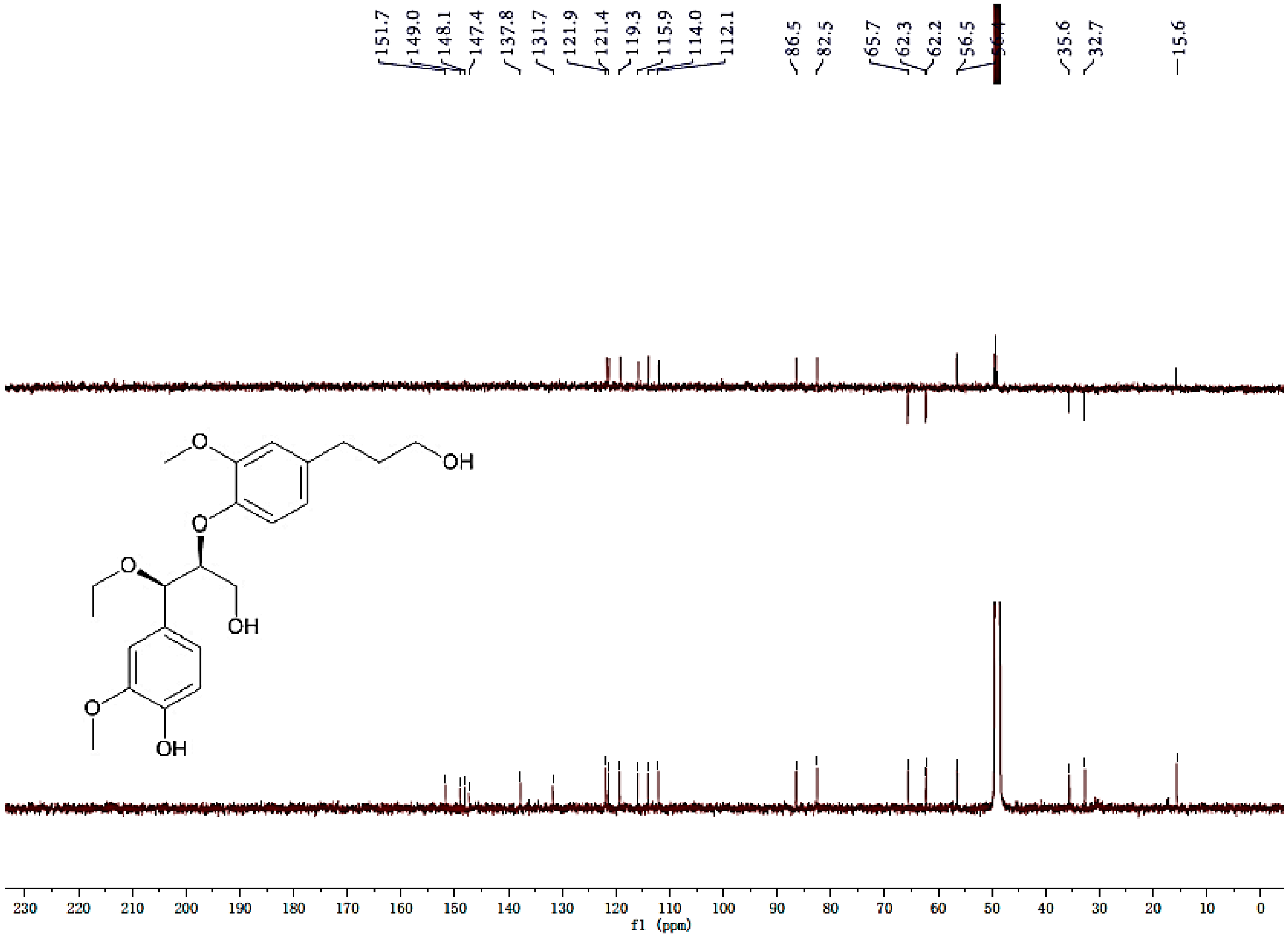

Figure S25. HSQC spectrum of compound 3 (3a/3b) in CD_3OD

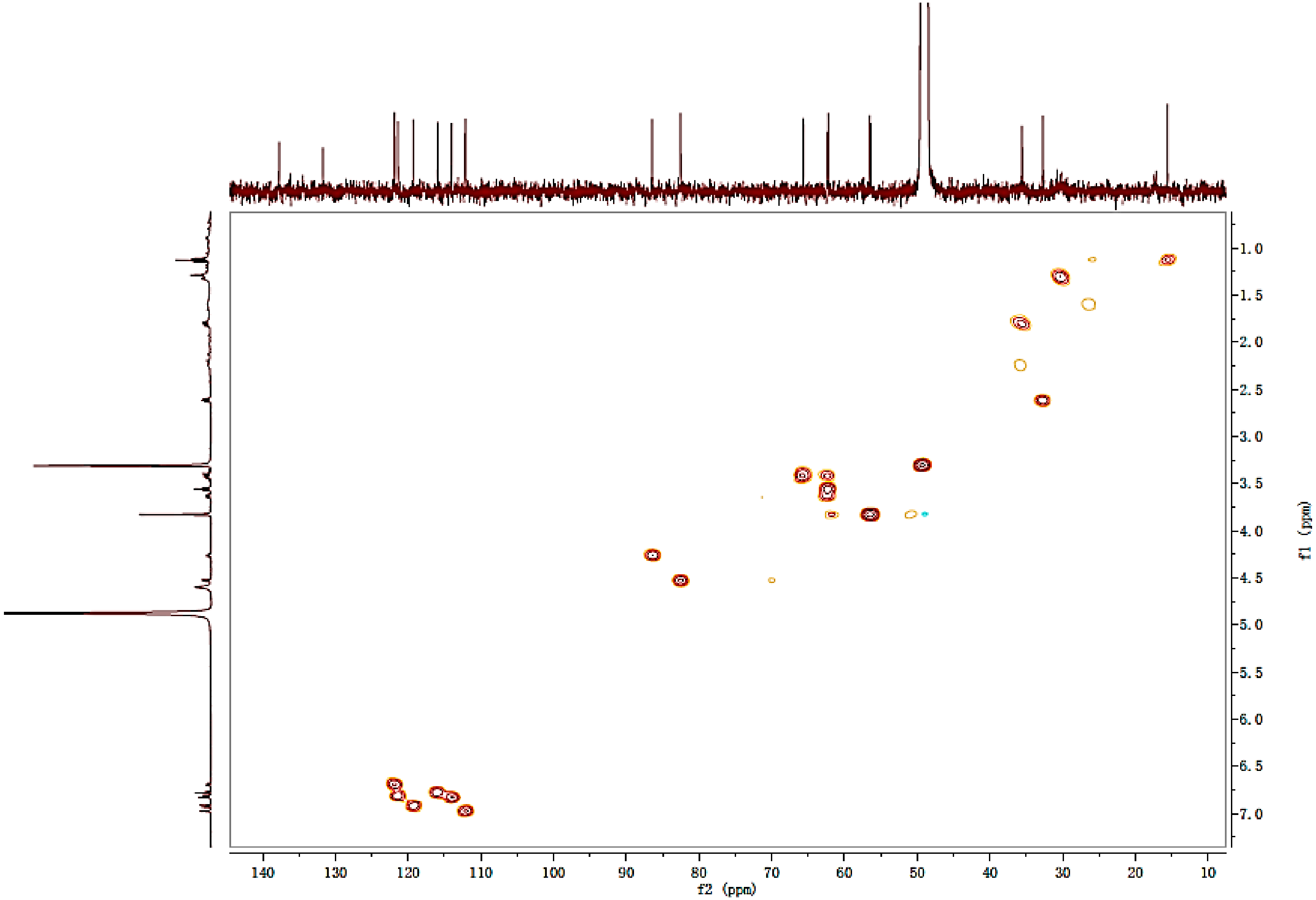

Figure S26. HMBC spectrum of compound 3 (3a/3b) in CD_3OD

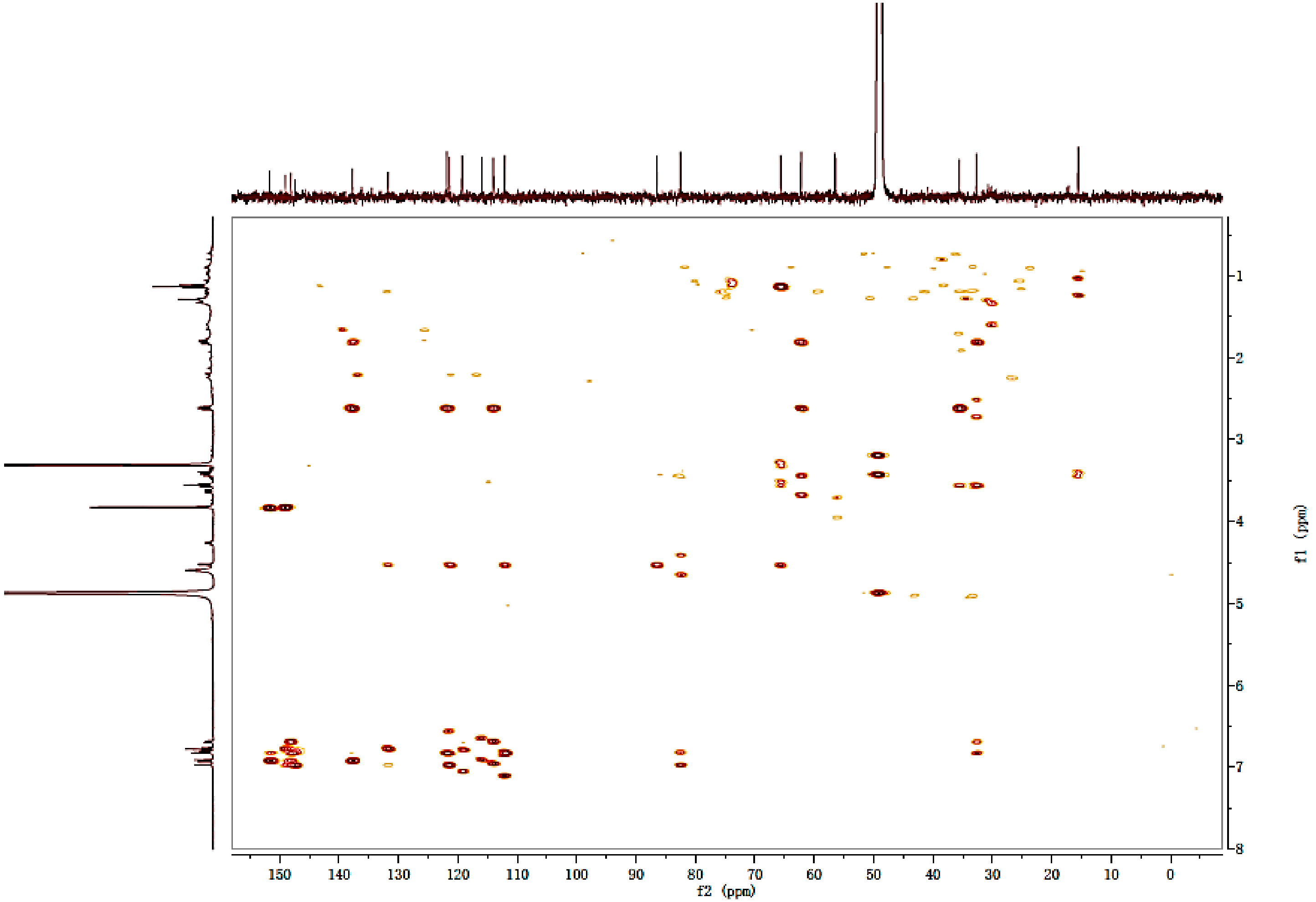

Figure S27. ^{1}H–^{1}H COSY spectrum of compound 3 (3a/3b) in CD_3OD

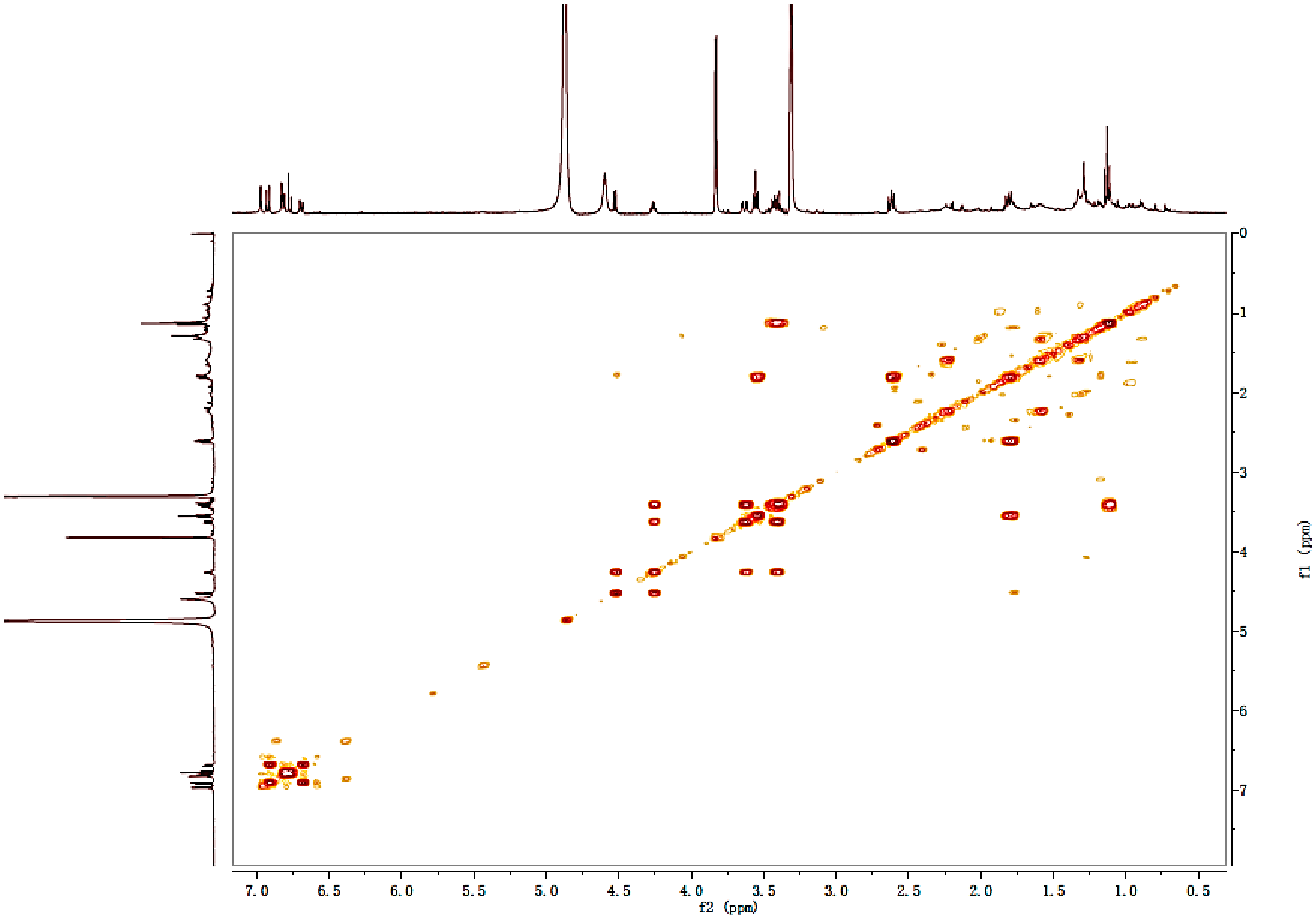

Figure S28. ROESY spectrum of compound 3 (3a/3b) in CD_3OD

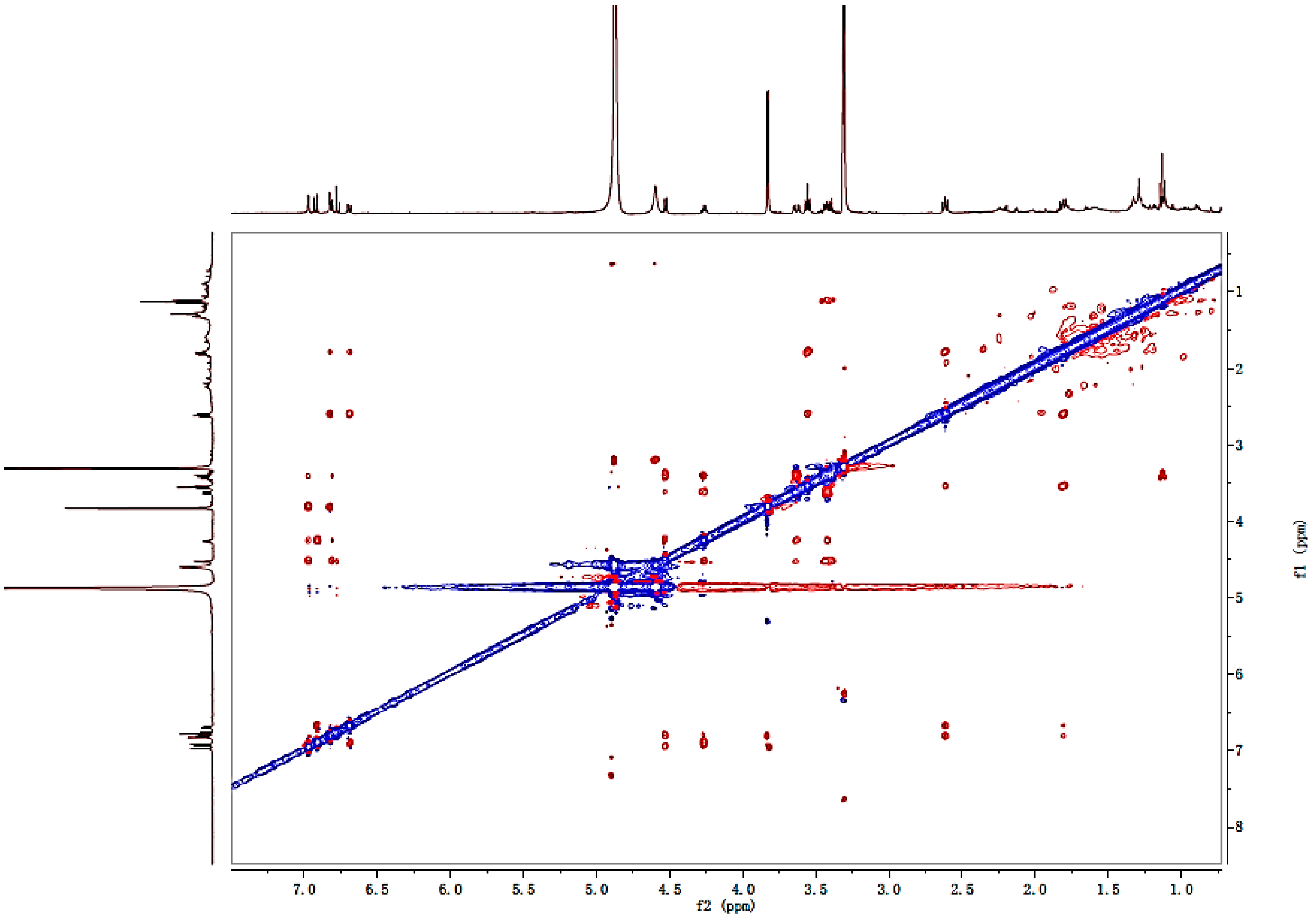

Figure S29. (+)-ESIMS spectrum of compound 3 (3a/3b)

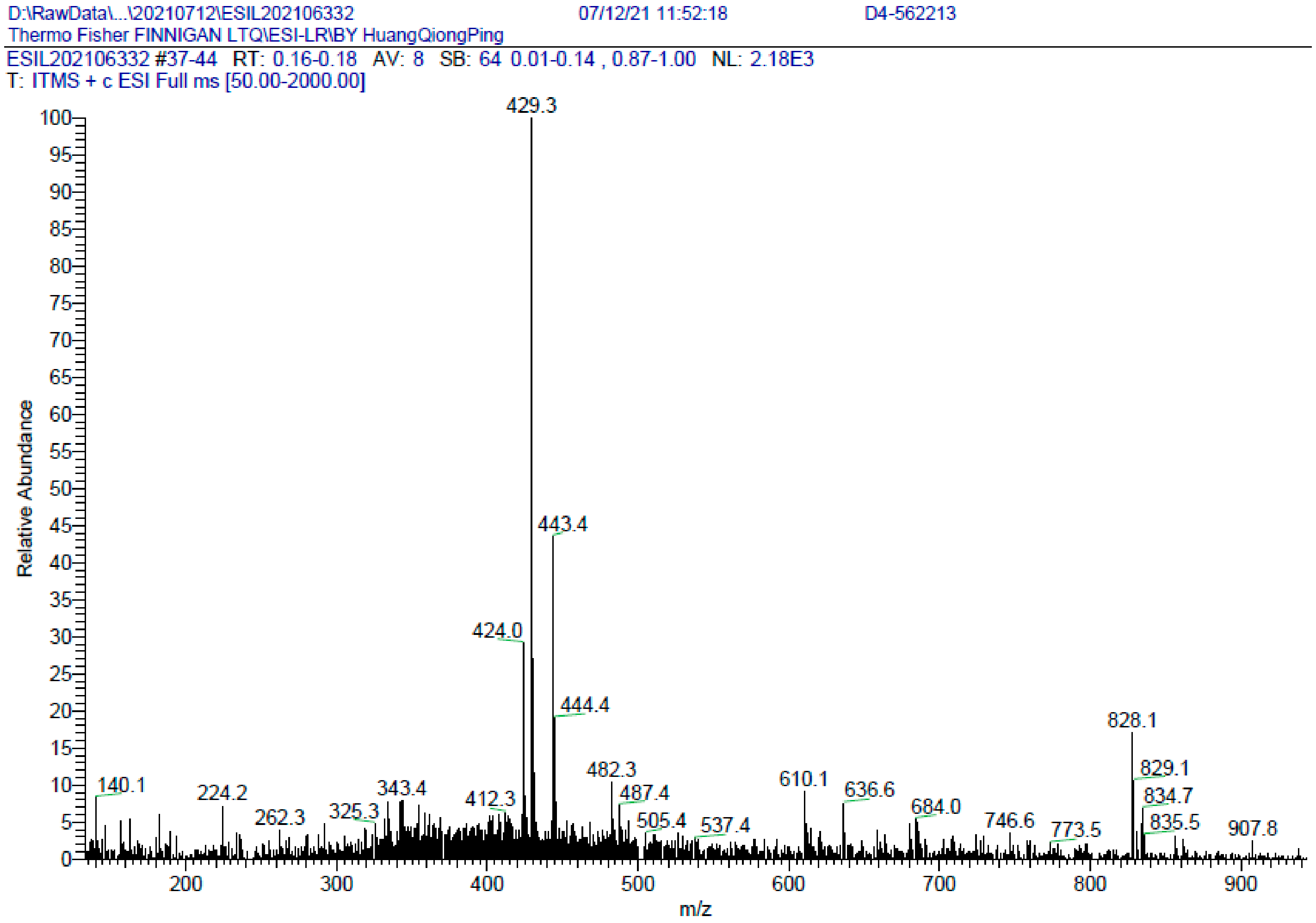

Figure S30. (-)-ESIMS spectrum of compound 3 (3a/3b)

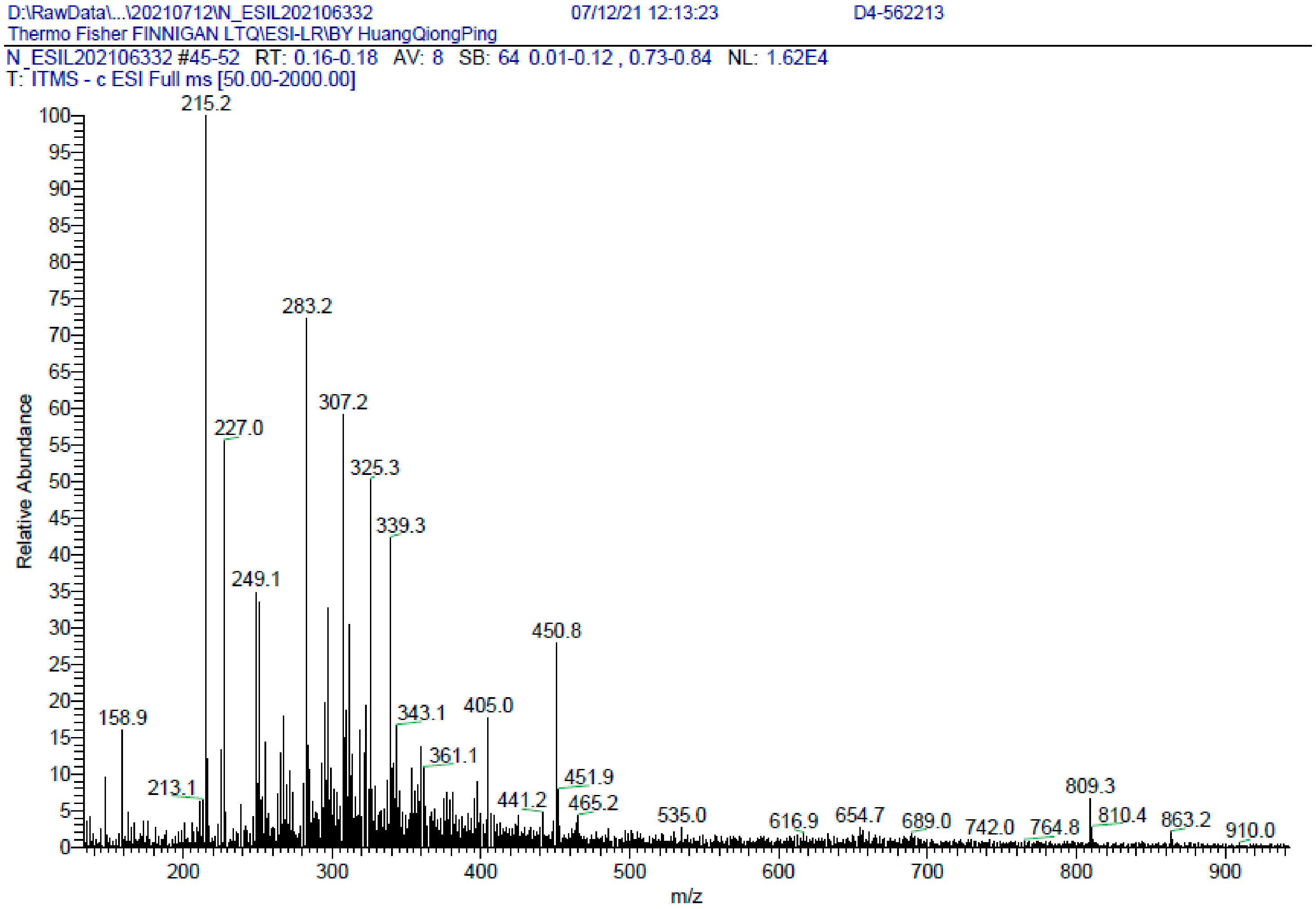

Figure S31. (+)-HRESIMS spectrum of compound 3 (3a/3b)

Qualitative Analysis Report

**Data Filename**	ESIH202103424.d	**Sample Name**	D4-562213
**Sample ID**		**Position**	P1-A5
**Instrument Name**	Agilent G6520 Q-TOF	**Acq Method**	20160322_MS_ESIH_POS_1min.m
**Acquired Time**	7/12/2021 18:13:22	**IRM Calibration Status**	Success
**DA Method**	small molecular data analysis method.m	**Comment**	ESIH by zhuzhenyun

User Spectra

**Fragmentor Voltage**	**Collision Energy**	**Ionization Mode**
135	0	ESI

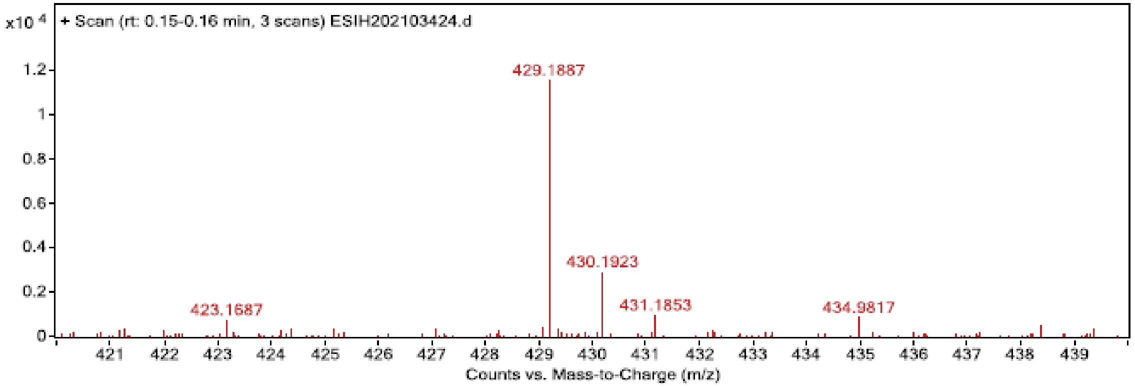

Formula Calculator Results

m/z	Calc m/z	Diff (mDa)	Diff (ppm)	Ion Formula	Ion
429.1887	429.1884	-0.33	-0.78	C22 H30 Na O7	(M+Na)+

--- End Of Report ---

Figure S32. IR spectrum of compound 3 (3a/3b)

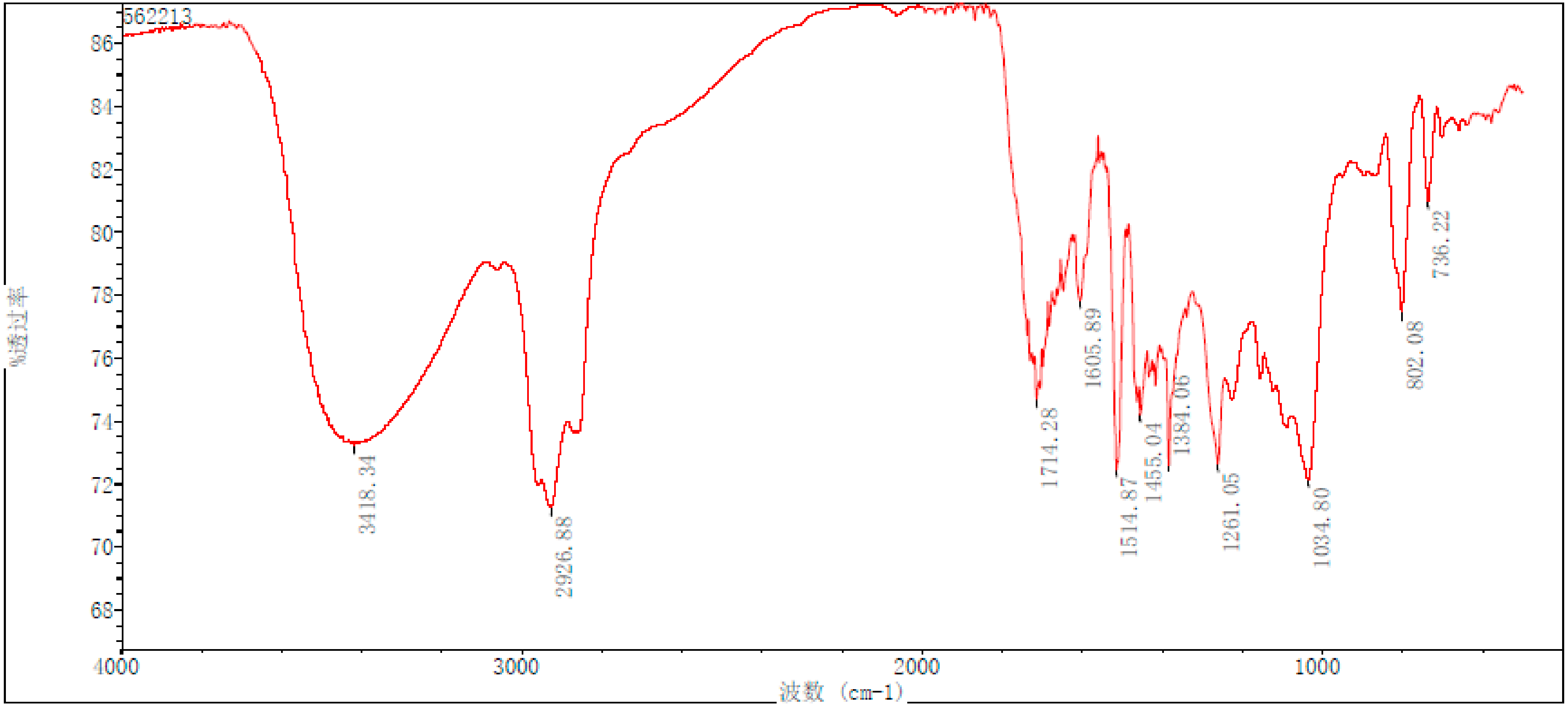

Figure S33. UV spectrum of compound 3 (3a/3b)

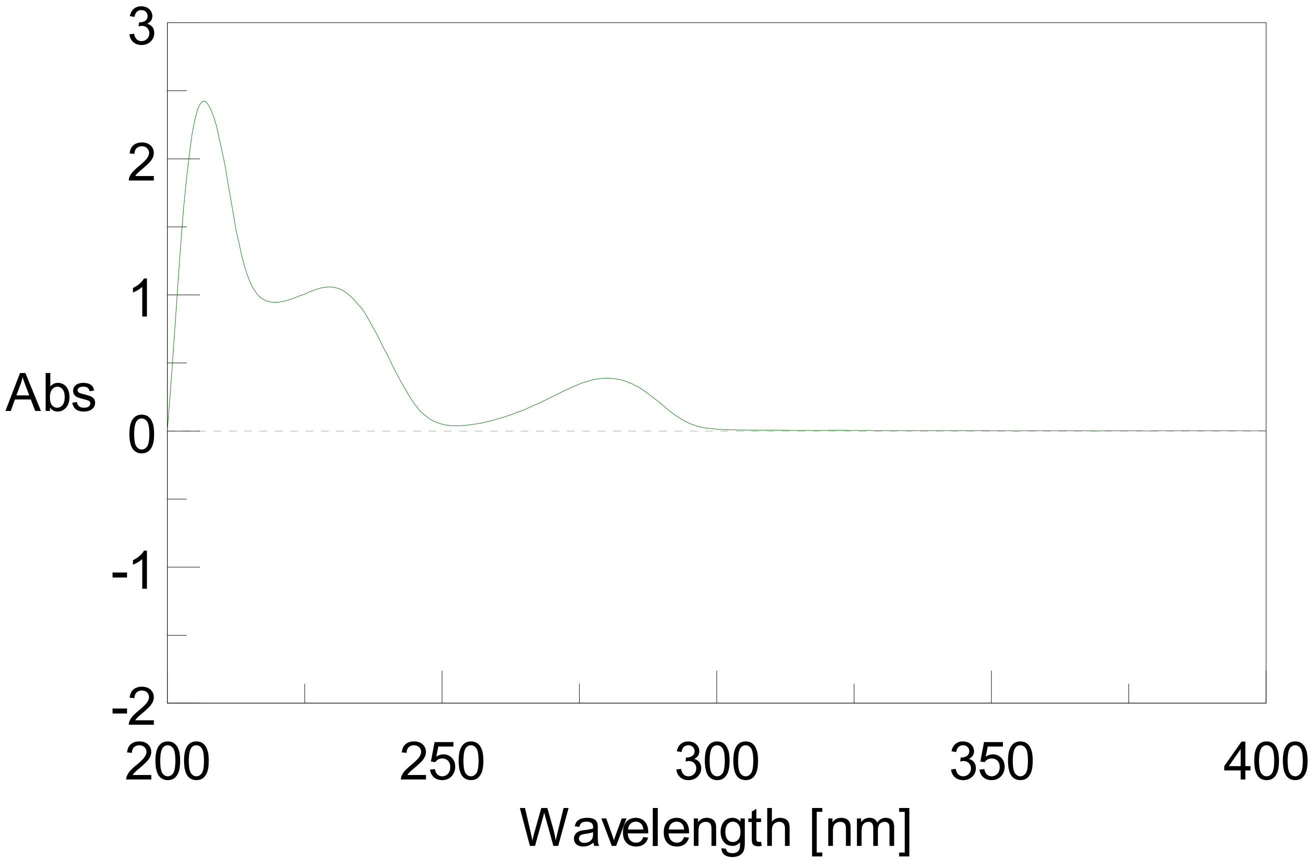

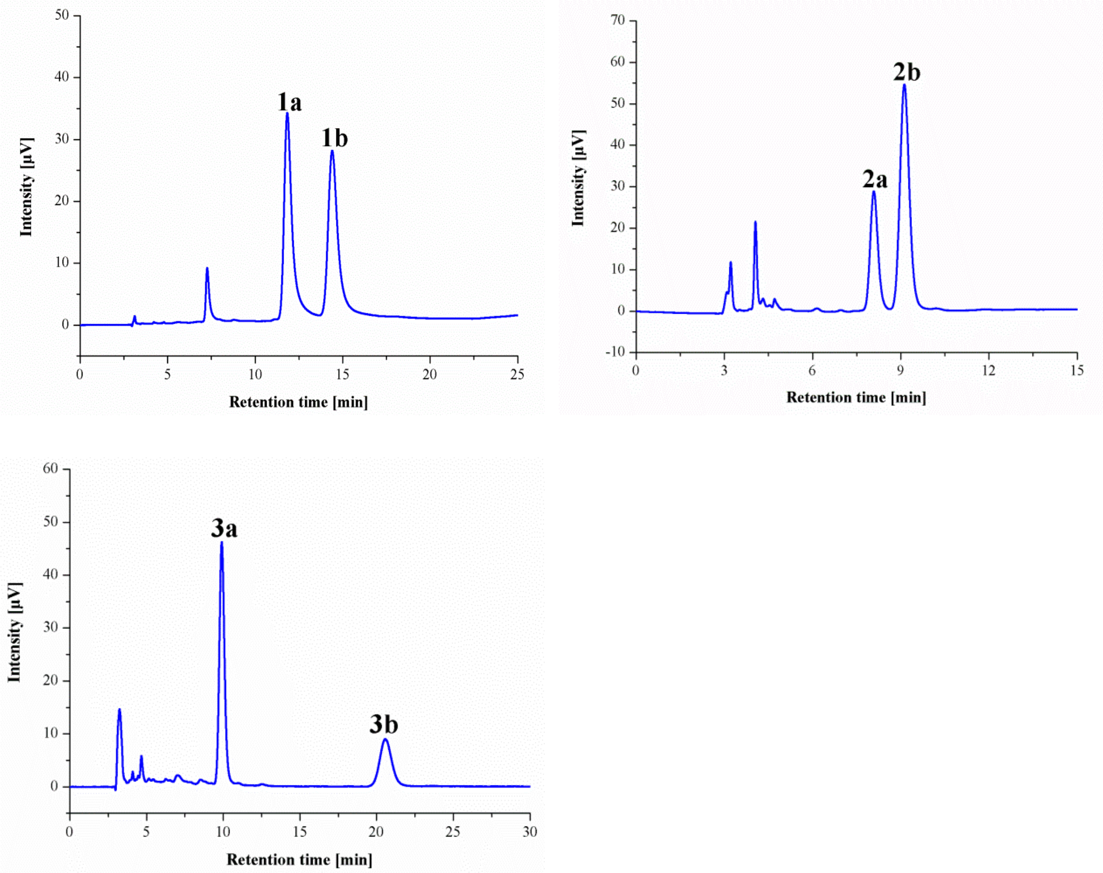

Figure S34. Chiral HPLC separation profiles of 1a/1b–3a/3b.

Figure S35. ^{1}H NMR spectrum of compound 4 in CD_3OD

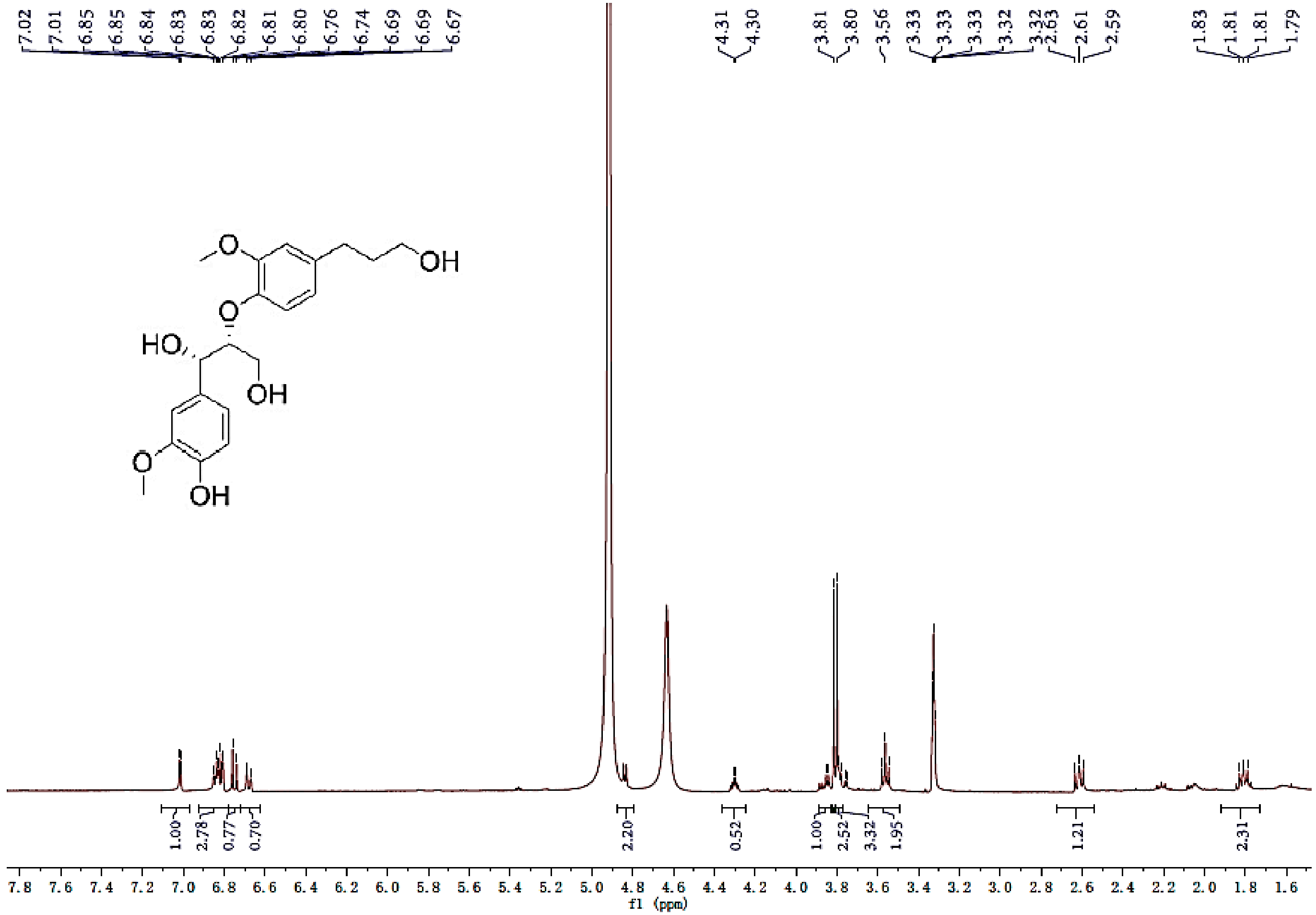

Figure S36. ^{13}**C NMR spectrum of compound 4 in** CD_3OD

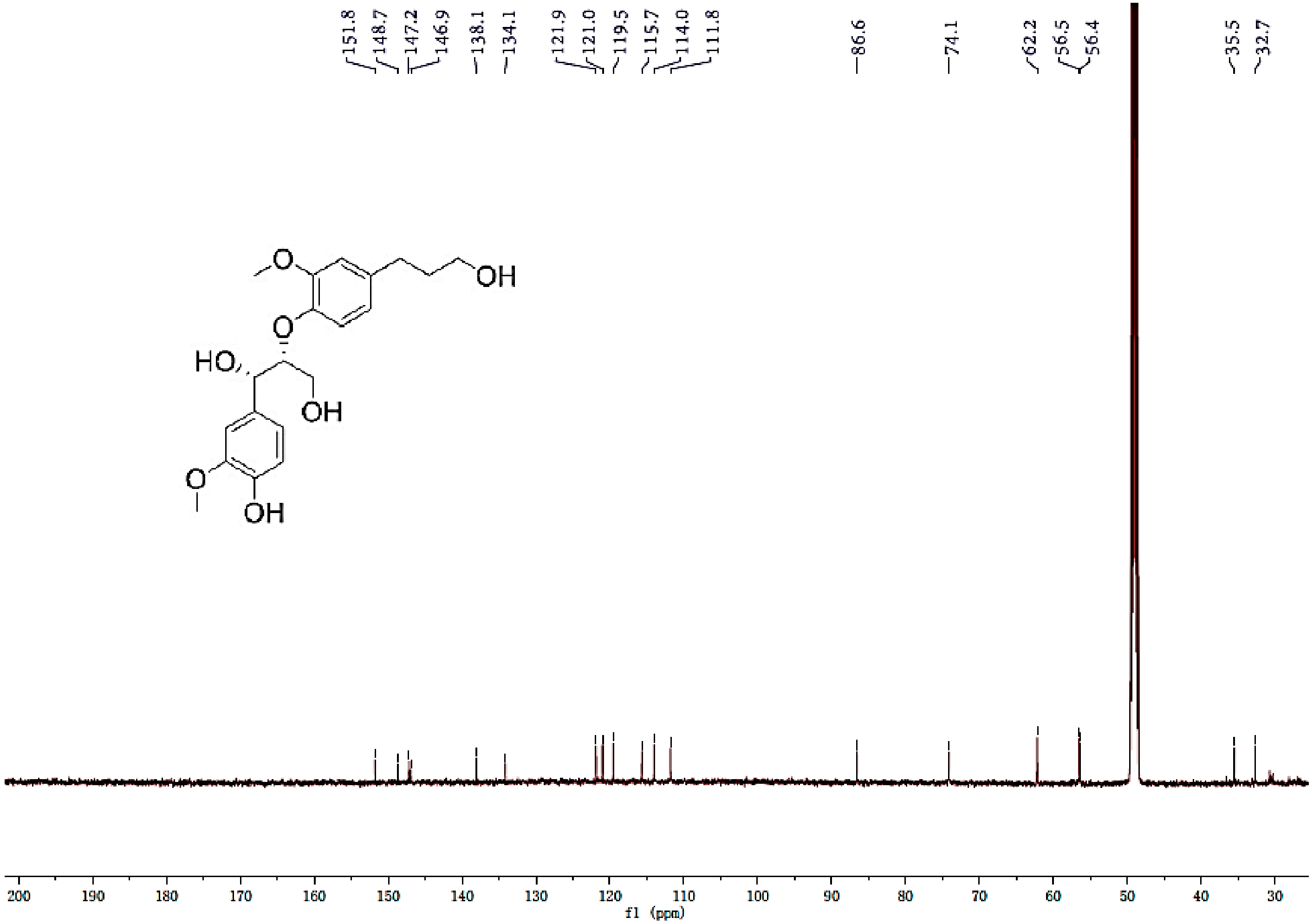

Figure S37. ^{1}H NMR spectrum of compound 5 in CD_3OD

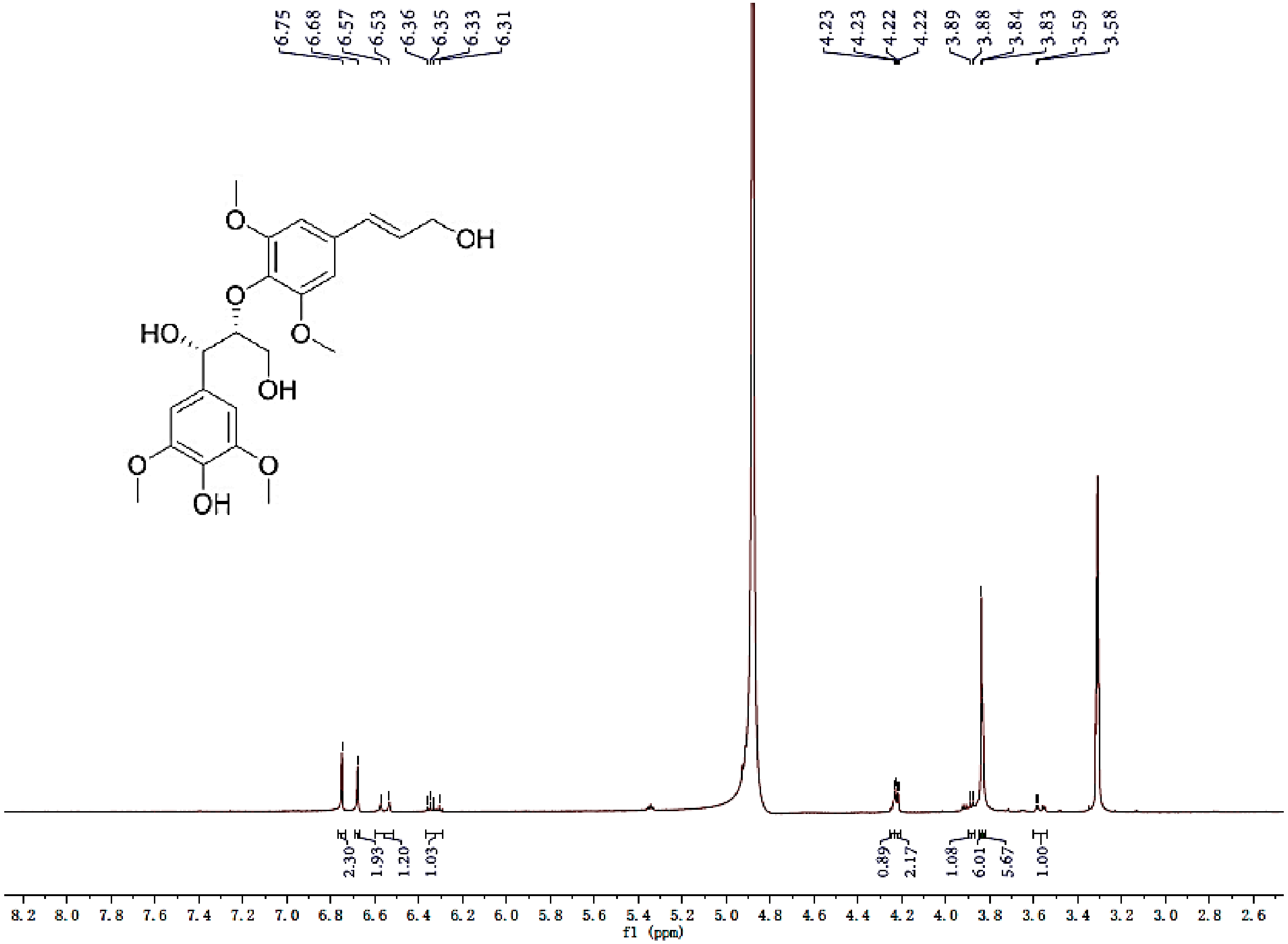

Figure S38. ^{13}**C NMR spectrum of compound 5 in** CD_3OD

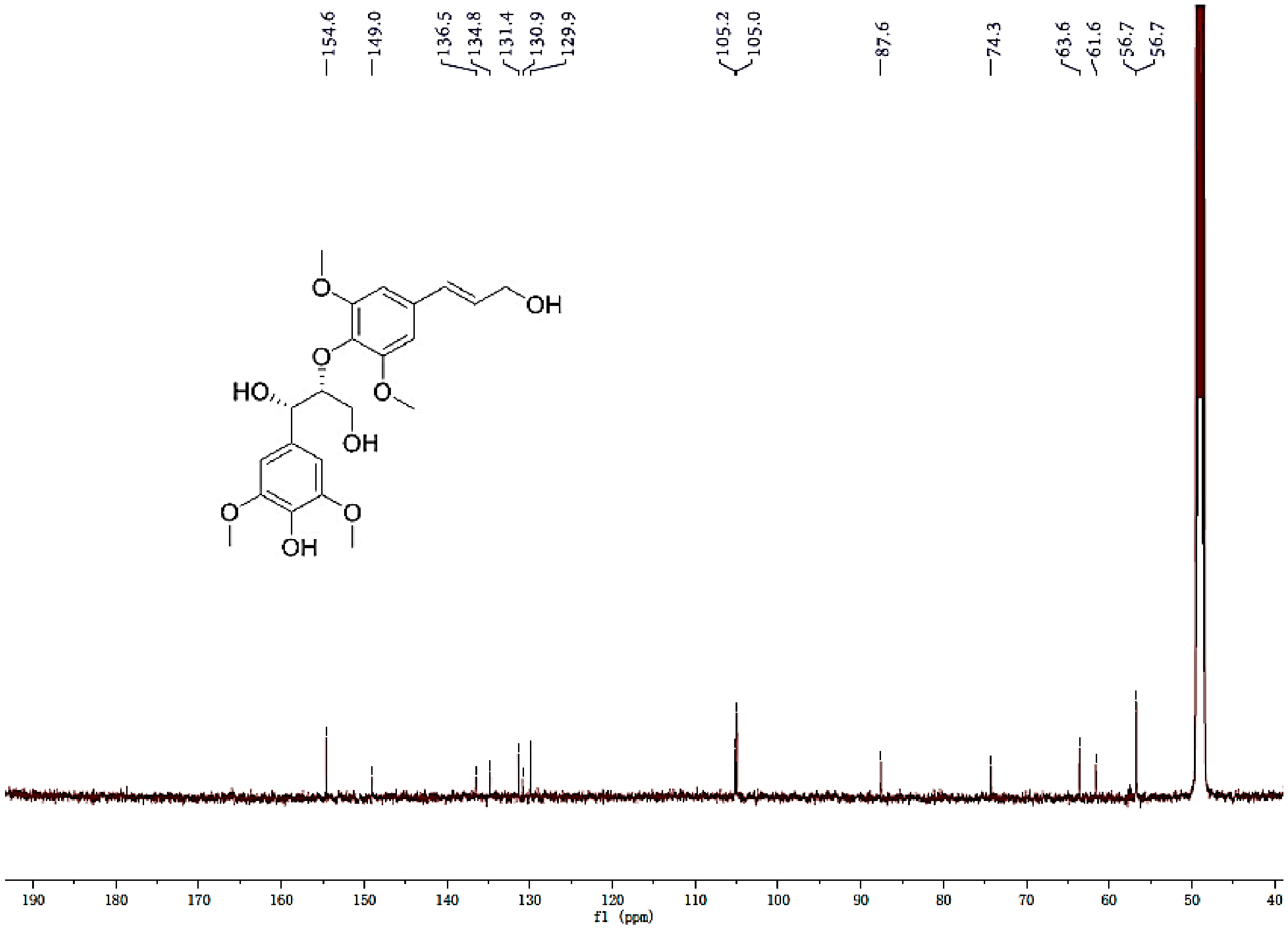

Figure S39. ^{1}H NMR spectrum of compound 6 in CD_3OD

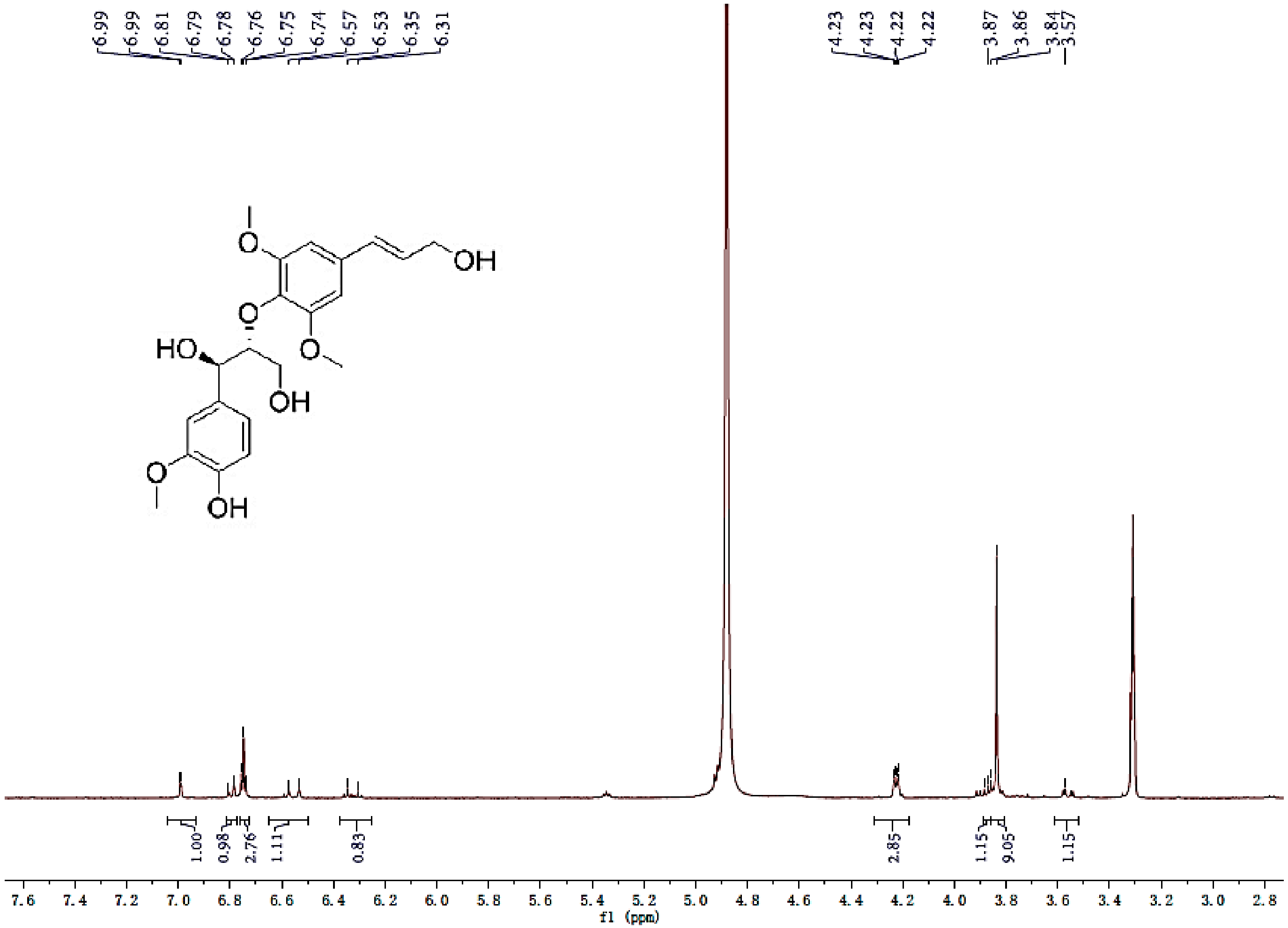

Figure S40. ^{13}C NMR spectrum of compound 6 in CD_3OD

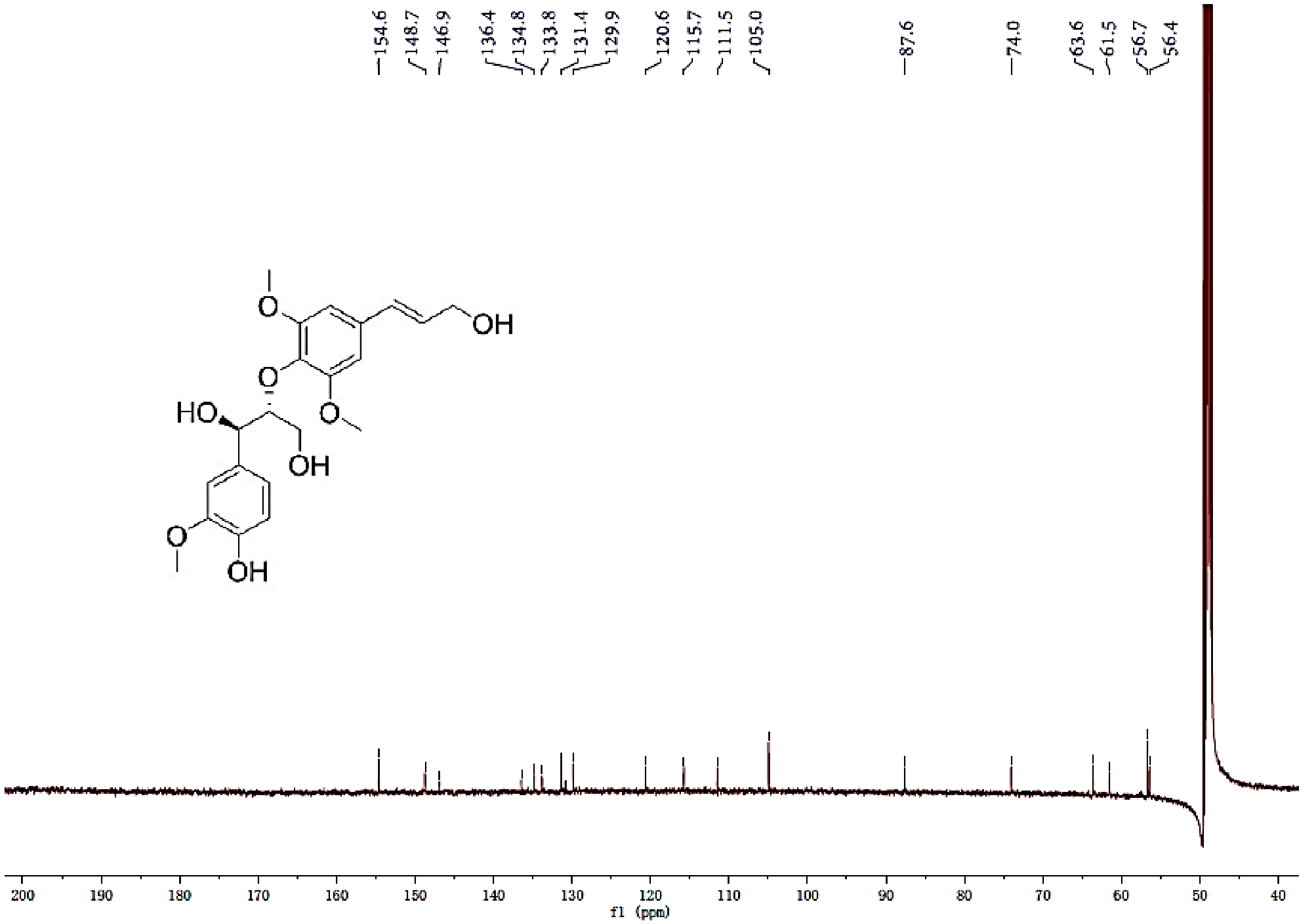

Figure S41. ^{1}H NMR spectrum of compound 7 in $CDCl_3$

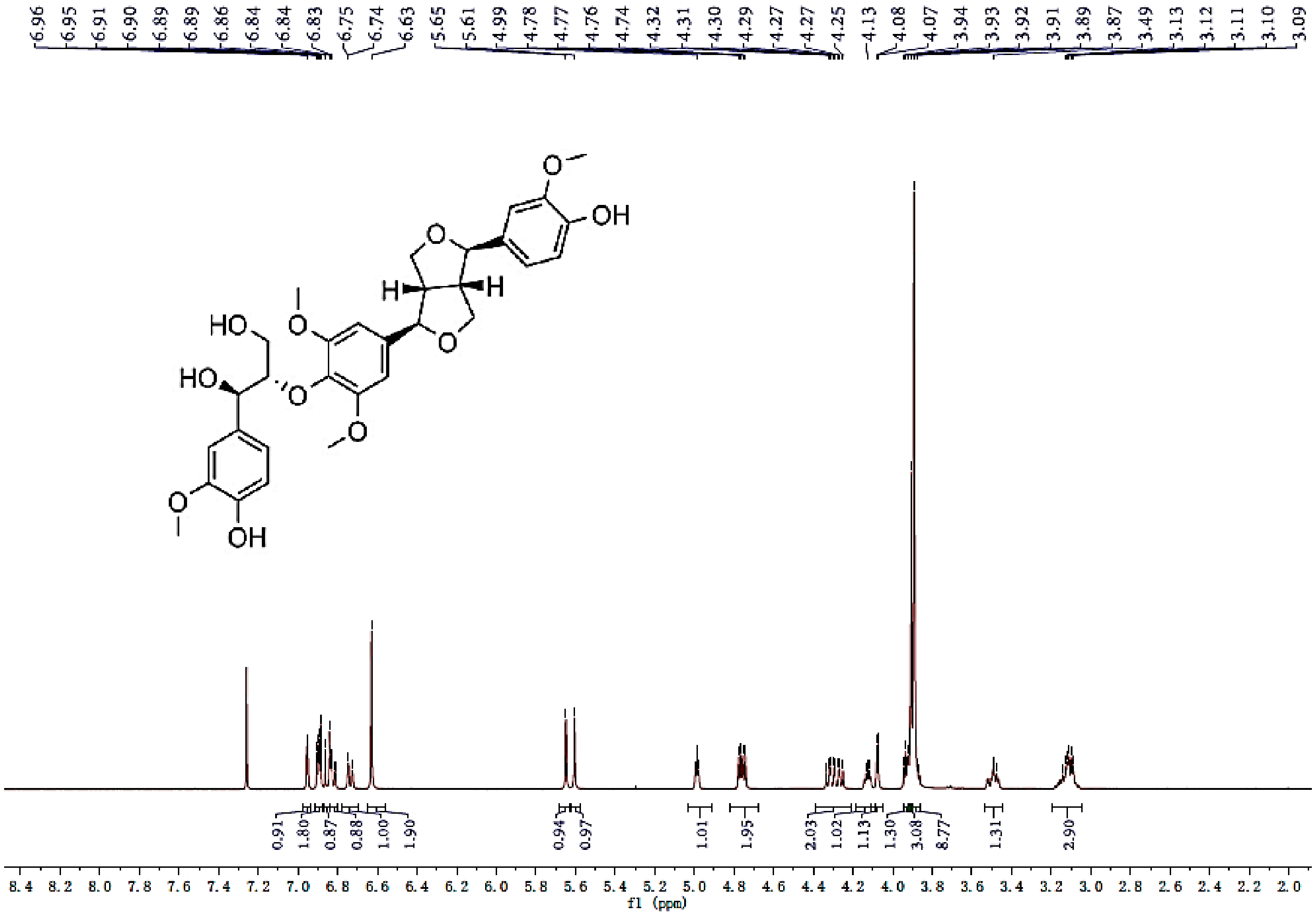

Figure S42. ^{13}C NMR spectrum of compound 7 in $CDCl_3$

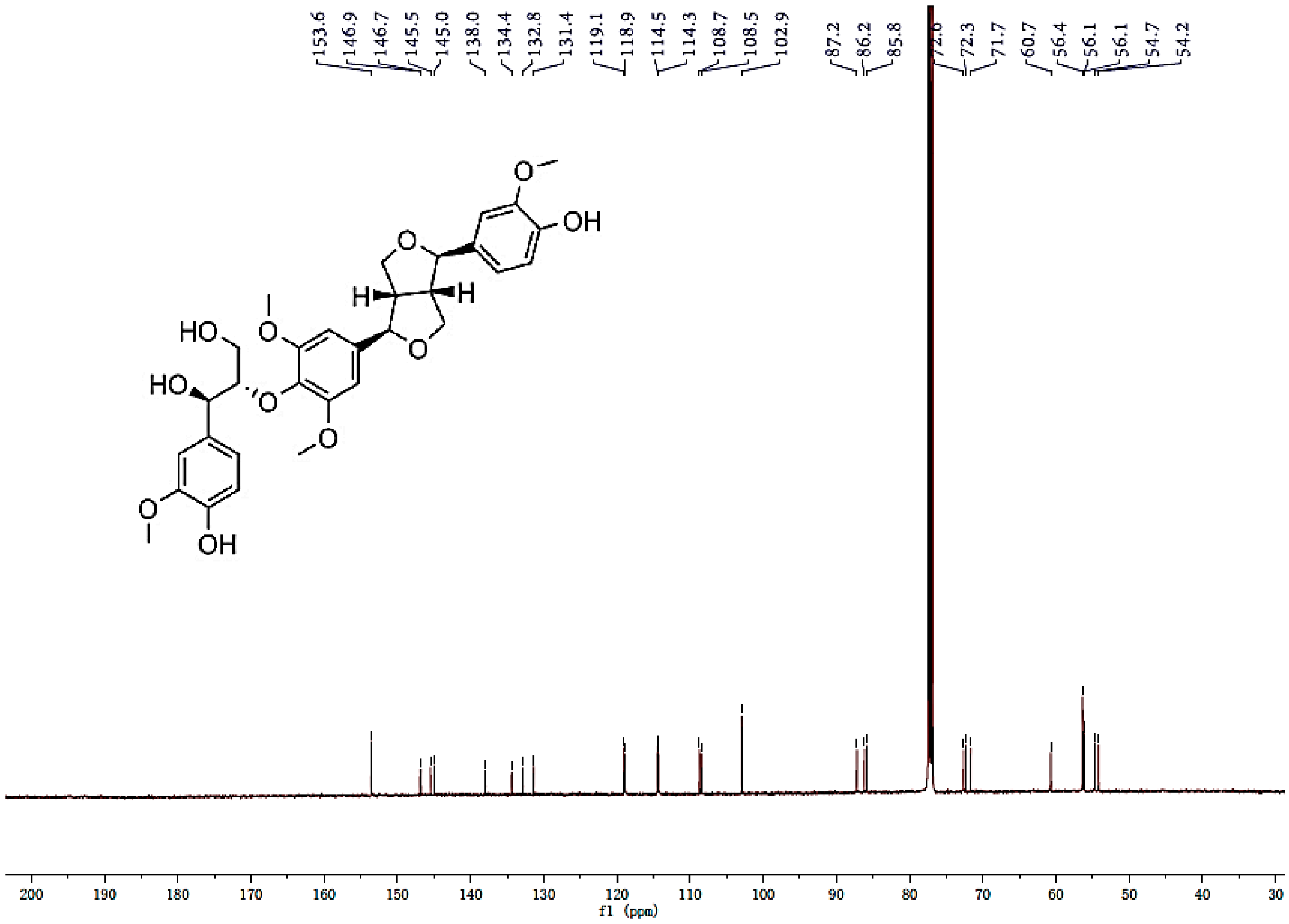

Figure S43. ^{1}**H NMR spectrum of compound 8 in CDCl$_3$**

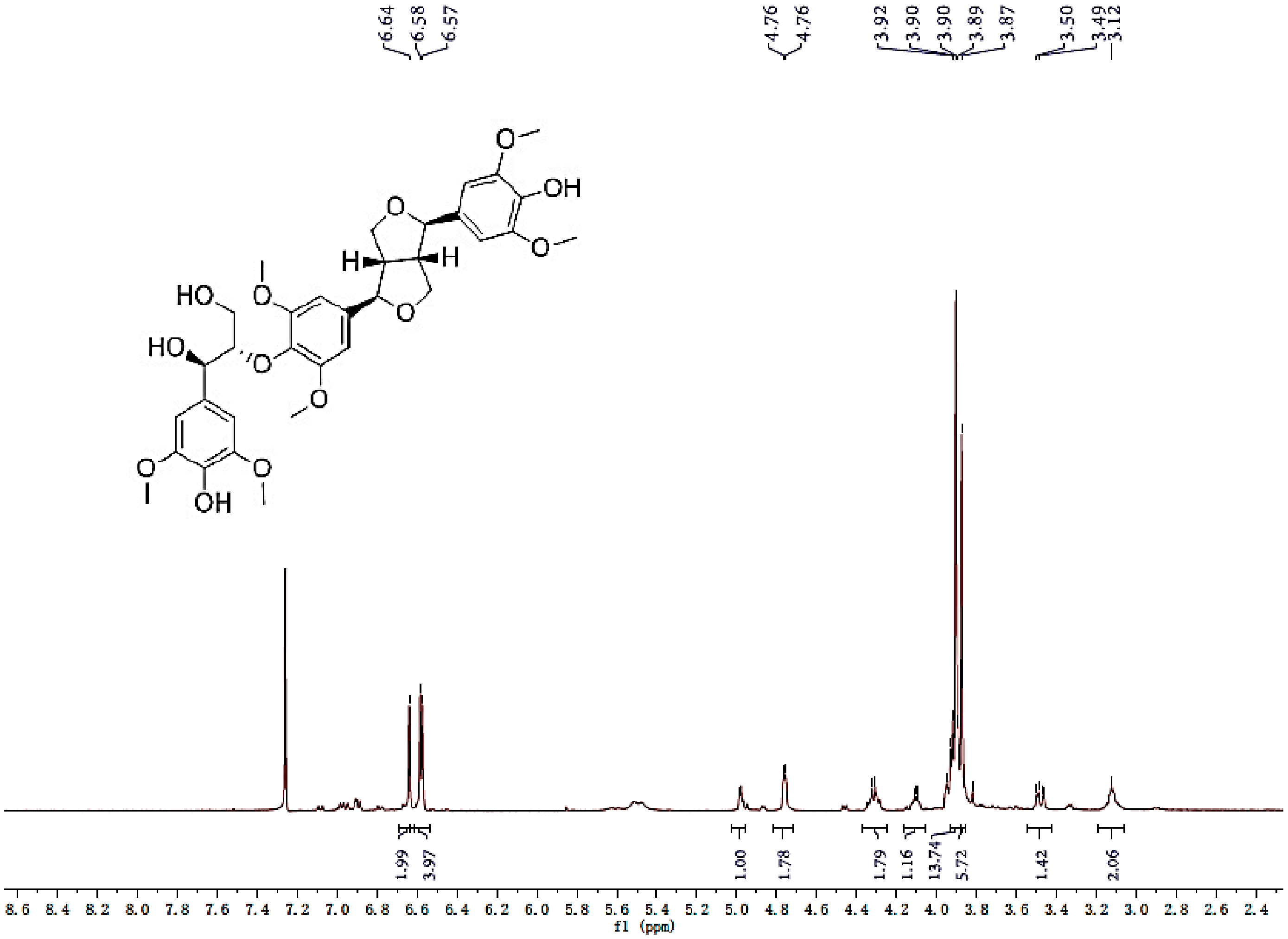

Figure S44. ^{13}C NMR spectrum of compound 8 in $CDCl_3$

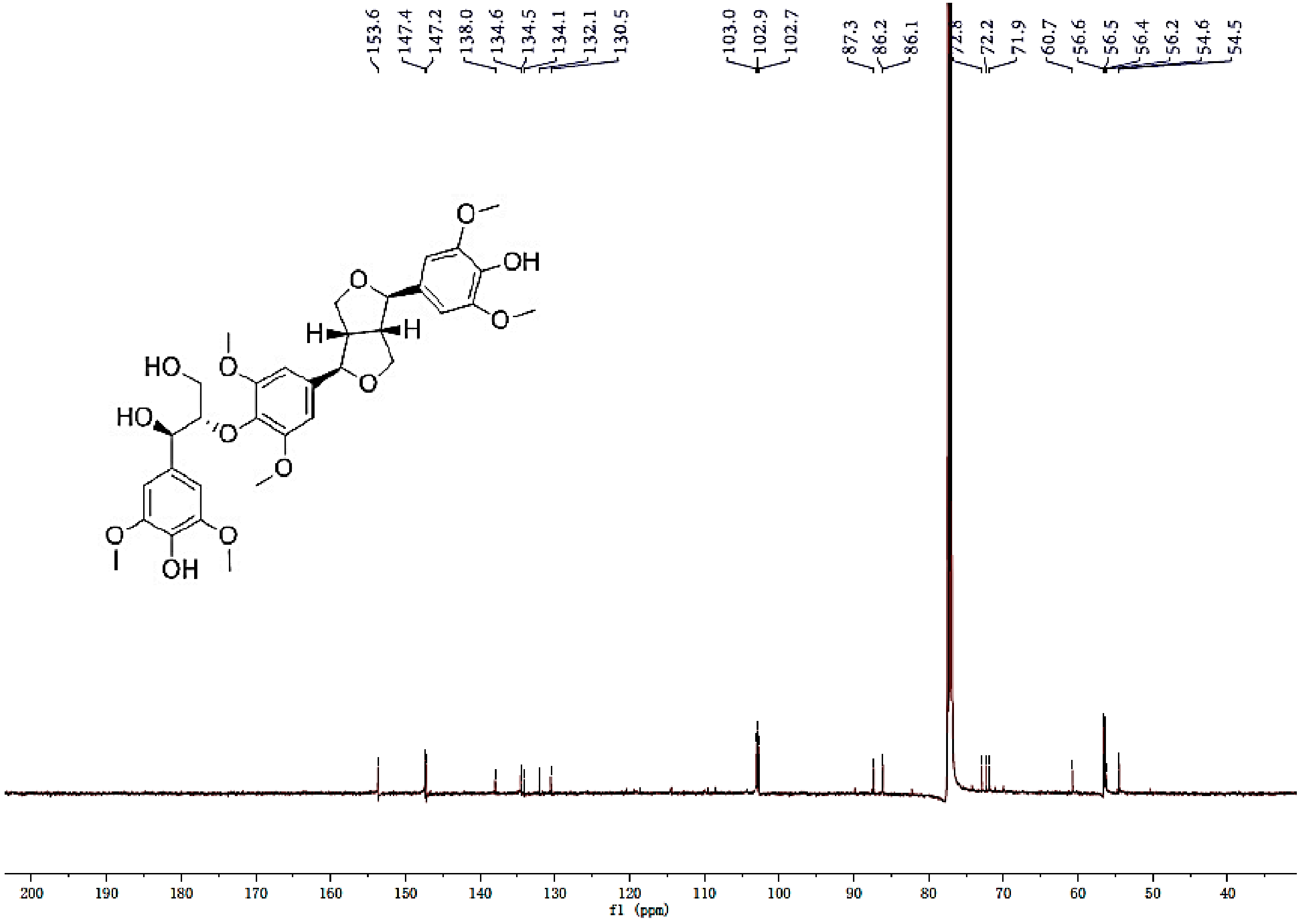

Figure S45. ^1H NMR spectrum of compound 9 in $CDCl_3$

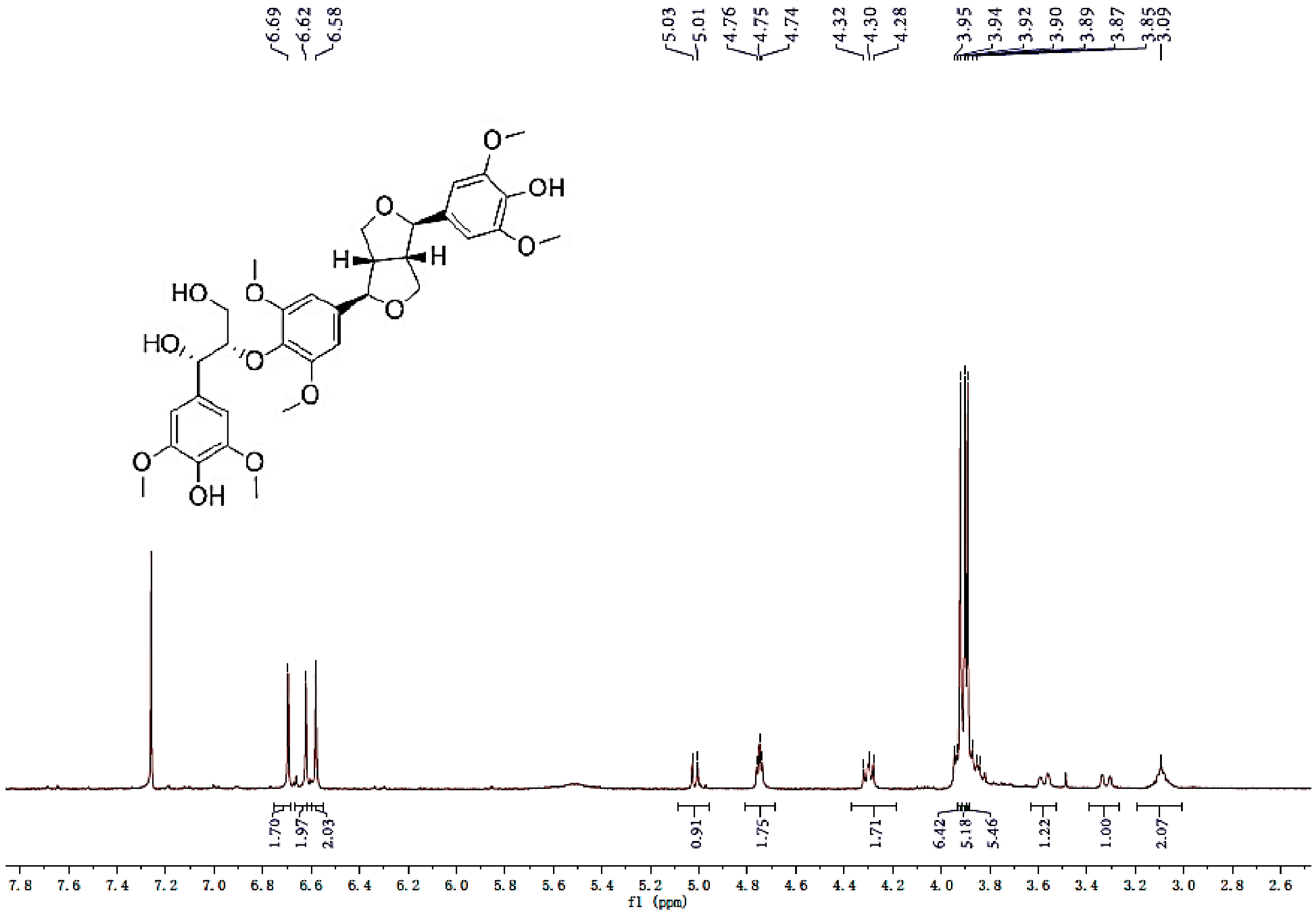

Figure S46. ^{13}C **NMR spectrum of compound 9 in** $CDCl_3$

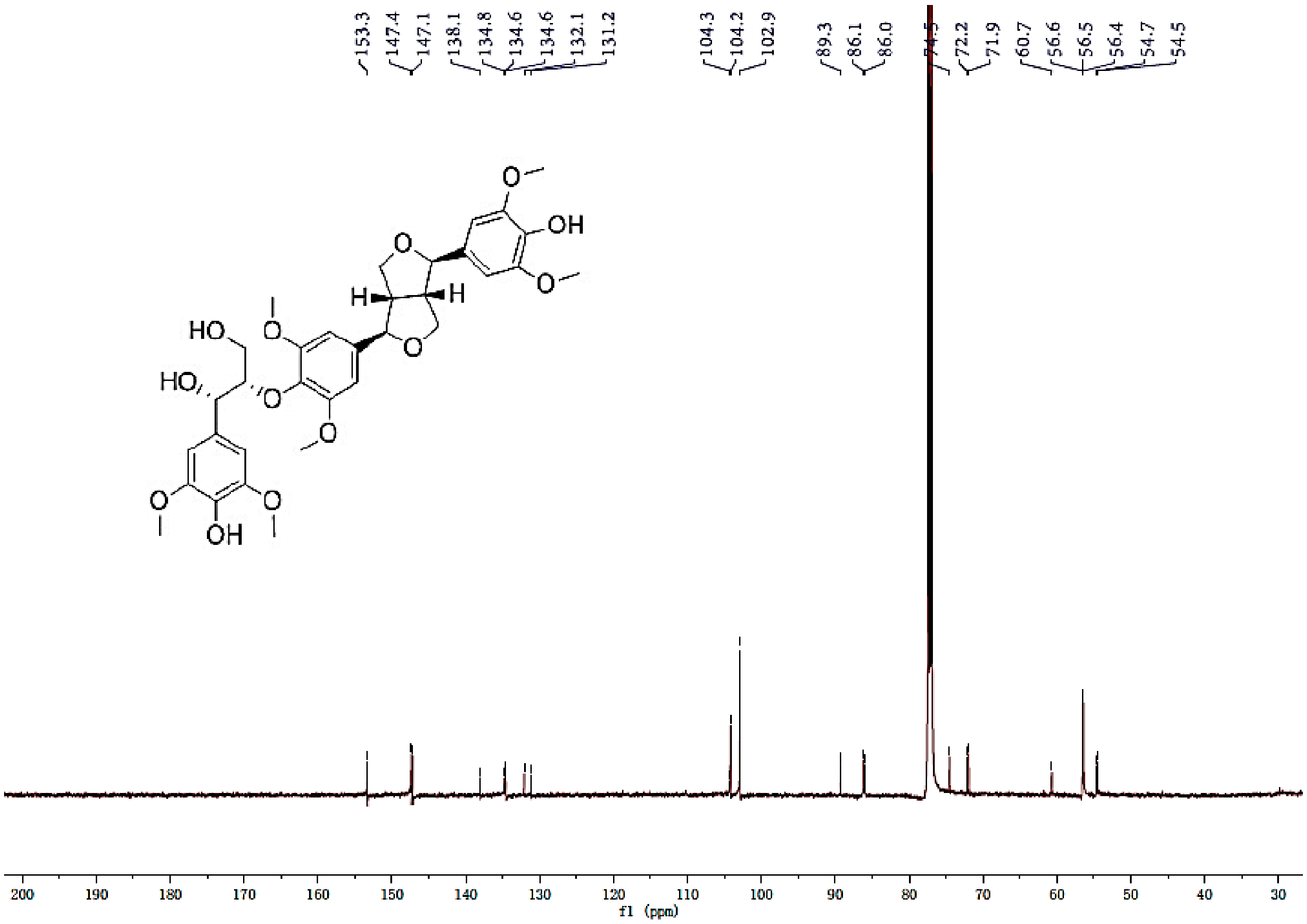

Figure S47. ^{1}H NMR spectrum of compound 10 in $CDCl_3$

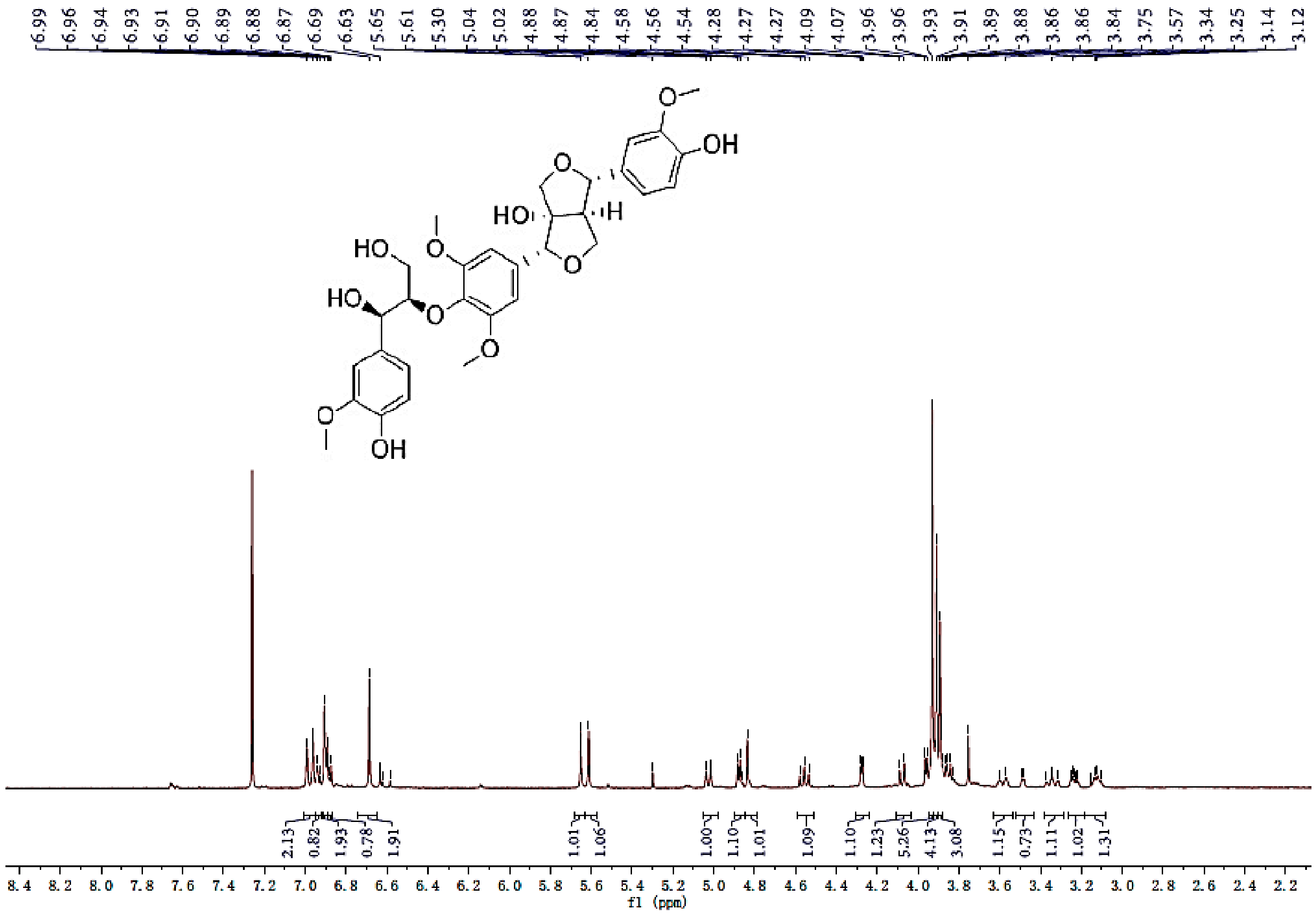

Figure S48. ^{13}C NMR spectrum of compound 10 in $CDCl_3$

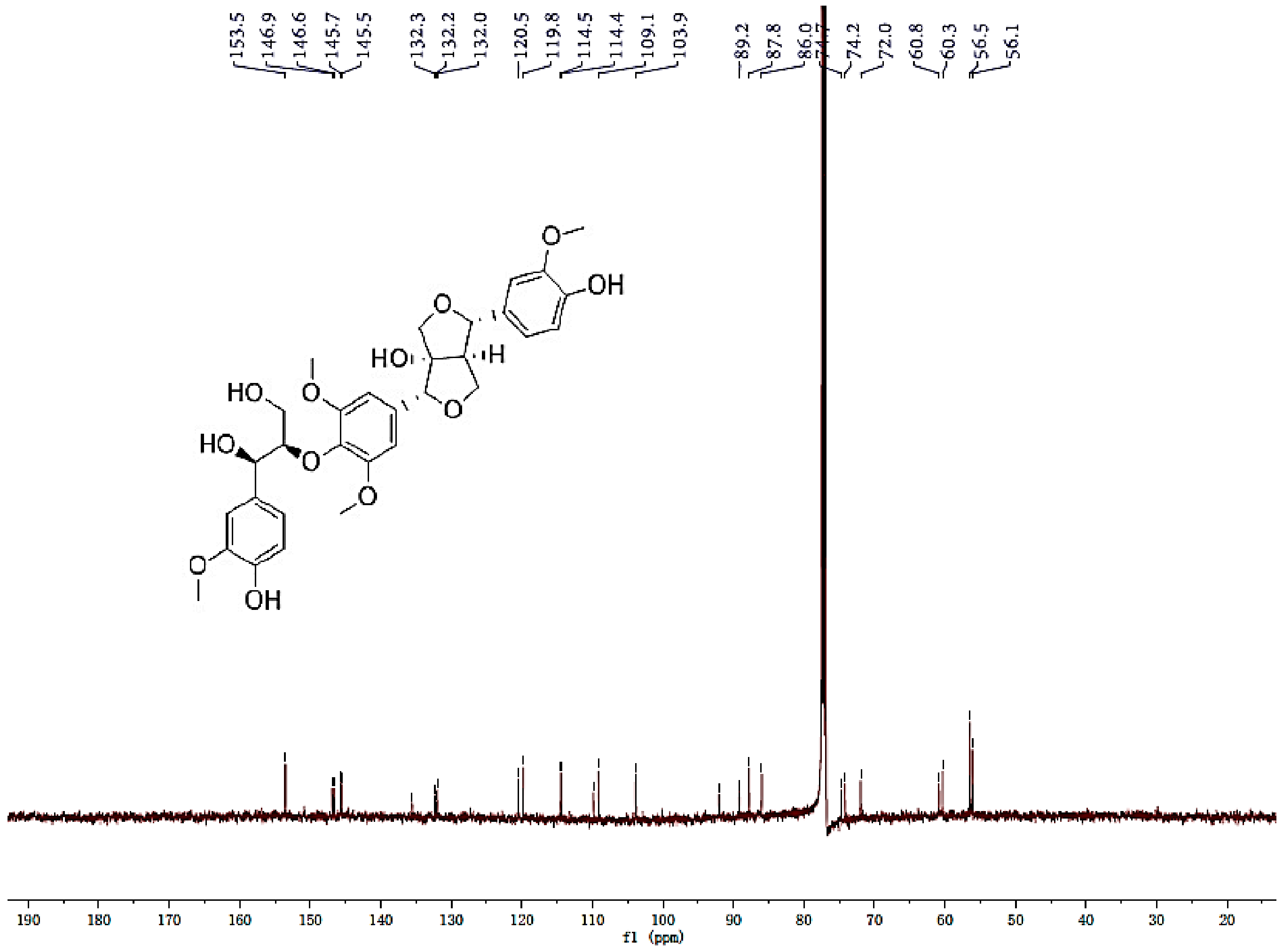

Supplement: Supplementary file 1 [file ijms-23-14062-s001.zip › ijms-1999944-supplementary.pdf]
